# Cost-effectiveness of the long-acting regimen cabotegravir plus rilpivirine for the treatment of HIV-1 and its potential impact on adherence and viral transmission: A modelling study

**DOI:** 10.1371/journal.pone.0245955

**Published:** 2021-02-02

**Authors:** Ben Parker, Tom Ward, Olivia Hayward, Ian Jacob, Erin Arthurs, Debbie Becker, Sarah-Jane Anderson, Vasiliki Chounta, Nicolas Van de Velde

**Affiliations:** 1 Health Economics and Outcomes Research Ltd, Pontprennau, Cardiff, United Kingdom; 2 Health Economics & Outcomes Research, GlaxoSmithKline, Toronto, Ontario, Canada; 3 Quadrant Health Economics Inc, Cambridge, Ontario, Canada; 4 Value Evidence and Outcomes, GlaxoSmithKline, Brentford, Middlesex, United Kingdom; 5 Global Health Outcomes, ViiV Healthcare Ltd, Brentford, Middlesex, United Kingdom; Boston University School of Public Health, UNITED STATES

## Abstract

**Introduction:**

Combination antiretroviral therapy (cART) improves outcomes for people living with HIV (PLWH) but requires adherence to daily dosing. Suboptimal adherence results in reduced treatment effectiveness, increased costs, and greater risk of resistance and onwards transmission. Treatment with long-acting (LA), injection-based ART administered by healthcare professionals (directly observed therapy (DOT)) eliminates the need for adherence to daily dosing and may improve clinical outcomes. This study reports the cost-effectiveness of the cabotegravir plus rilpivirine LA regimen (CAB+RPV LA) and models the potential impact of LA DOT therapies.

**Methods:**

Parameterisation was performed using pooled data from recent CAB+RPV LA Phase III trials. The analysis was conducted using a cohort-level hybrid decision-tree and state-transition model, with states defined by viral load and CD4 cell count. The efficacy of oral cART was adjusted to reflect adherence to daily regimens from published data. A Canadian health service perspective was adopted.

**Results:**

CAB+RPV LA is predicted to be the dominant intervention when compared to oral cART, generating, per 1,000 patients treated, lifetime cost-savings of $1.5 million, QALY and life-year gains of 107 and 138 respectively with three new HIV cases averted.

**Conclusions:**

Economic evaluations of LA DOTs need to account for the impact of adherence and HIV transmission. This study adds to the existing literature by incorporating transmission and using clinical data from the first LA DOT regimen. Providing PLWH and healthcare providers with novel modes of ART administration, enhancing individualisation of treatment, may facilitate the achievement of UNAIDS 95-95-95 objectives.

## Introduction

Human Immunodeficiency Virus (HIV) and its clinical manifestation of Acquired Immune Deficiency Syndrome (AIDS) represent a significant global economic, clinical and humanistic burden [1, 2]. Despite improvements in treatment access and advances in antiretroviral therapies (ARTs) [1], over 35 million people were living with HIV/AIDS worldwide in 2019 with over 690,000 deaths attributed to HIV-related illnesses [2, 3]. In 2014, a 95-95-95 target was set by the United Nations Programme on HIV/AIDS (UNAIDS) for 2030, aiming to diagnose 95% of HIV positive people, provide ART to 95% of those diagnosed and for 95% of those treated to achieve virologic suppression [4].

Virologic suppression, the primary goal of ART, leads to an improvement in quality of life, reduces HIV related morbidity and mortality [5] and significantly reduces the risk of viral transmission [6, 7]. Adherence to daily ART, however, is essential for ensuring virologic suppression in people living with HIV (PLWH) [8, 9]. The control and prevention of HIV infection therefore relies largely upon treatment adherence to achieve optimal HIV outcomes [10]. Poor adherence has been demonstrated as a major determinant of virologic failure, drug resistance, disease progression, hospitalisations, mortality and health care costs, as well as increasing the risk of onwards viral transmission [9, 11]. Treatments which improve adherence, whilst maintaining the low viral load levels achieved by current ART therapies, are therefore likely to help in reducing HIV transmissions [12].

Non-adherence to ART regimens has multiple causes, with pill burden, dosing frequency, dietary restrictions, HIV-related stigma and side effects most commonly reported [8, 13, 14]. Improving adherence by reducing pill burden [9], using long-acting (LA) regimens [15] or directly observed therapies (DOT) where a health care professional (HCP) is present during treatment administration [16], may hold significant potential in reducing the global burden of disease. Furthermore, increasing patient choice by providing alternative regimens with different modes of administration may increase the proportion of PLWH receiving and adhering to treatment, contributing to the UNAIDS 95-95-95 target to eradicate HIV [17, 18].

Cabotegravir plus rilpivirine LA (CAB+RPV LA) is a complete HIV regimen administered by intramuscular (IM) injection consisting of LA formulations of the integrase strand transfer inhibitor (INSTI) CAB and the non-nucleoside reverse-transcriptase inhibitor (NNRTI) RPV [19]. The treatment is indicated for virologically-suppressed adults with HIV-1 and in clinical trials has been administered either monthly or every two months. The regimen is administered by an HCP and is a DOT, providing patients and HCPs with the reassurance that the treatment has been administered correctly and at the right time. Phase III trials have shown noninferior efficacy compared to standard of care (SoC) oral ART, with the injections generally well-tolerated [20]. Trial participants reported significantly increased treatment satisfaction and preference for CAB+RPV LA compared to daily oral therapies [21, 22].

Cost-effectiveness analyses have estimated the public health benefits of interventions improving suboptimal adherence to ART [23]. The impact of LA ART on adherence was assessed by Ross et al. [15] which demonstrated that by eliminating the need for adherence to daily dosing, LA ART could provide significant health benefits compared to current daily oral ART when targeting a poorly adherent population. A recognised limitation of the study, however, was that it did not account for onwards viral transmission. Based on data from recent Phase III trials, this study aims to estimate the cost-effectiveness of CAB+RPV LA taking both adherence and viral transmission into account. Scenario and sensitivity analyses have been conducted to illustrate the impact of model assumptions.

## Methods

### Cost-effectiveness model

A previously presented and validated hybrid decision-tree and Markov model [24–26] was adapted to estimate the incremental costs and health outcomes associated with improved adherence among HIV-infected, virologically-supressed patients in Canada. A lifetime horizon (to age 100) and one-month cycle length were utilised, in order to capture patients’ whole life expectancy and provide sufficient granularity to capture clinical changes. Literature reviews to inform model parameters were conducted between March and April 2018. The model was designed and implemented in Microsoft Excel for Windows 2010 (Microsoft Corp, Redmond, CA, USA). Analysis of the transitions was performed in WinBugs v1.4.3 August 6^th^ 2007 (BUGS Project, Cambridge, UK).

**Fig 1:**
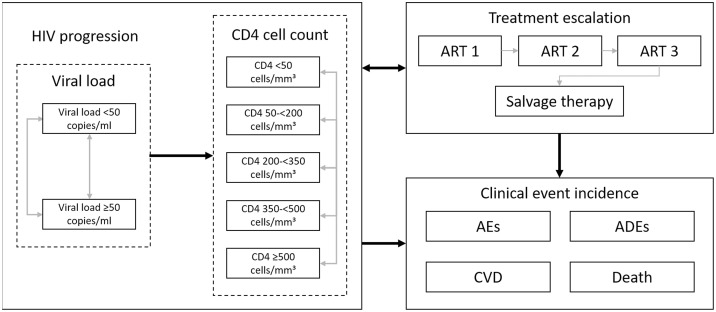
Model flow diagram. Abbreviations: ADE: AIDS defining event; AE: adverse event; ART: antiretroviral therapy; CD4: cluster of differentiation 4; CVD: cardiovascular disease.

Fig 1 outlines the treatment pathways and health states modelled. Health states reflect treatment line, virologic response and cluster of differentiation 4 (CD4) cell count. Patients can experience AIDS-defining events (ADEs), treatment-related adverse events and cardiovascular disease (CVD). Patients can discontinue to subsequent treatment lines for virologic or non-virologic reasons. The model structure, inputs and validation are described in detail in S1 Appendix.

### Clinical parameters

Clinical parameters were pooled from the primary analysis population (intent-to-treat exposed [ITT-E]) from the ATLAS (n = 283; NCT02951052) and FLAIR (n = 308; NCT02938520) trials [21, 22, 27], with sample sizes facilitating a >90% power to assess noninferiority. ATLAS, ‘Antiretroviral Therapy as Long Acting Suppression’ and FLAIR, ‘First Long-Acting Injectable Regimen’, are Phase III, multicentre, parallel-group, non-inferiority trials evaluating whether ART-suppressed HIV-1 patients remain suppressed after switching from their current regimen to CAB+RPV LA. ATLAS evaluated patients who were on SoC ART (two nucleoside reverse-transcriptase inhibitors [NRTIs] + a third agent) for a median time of 4.3 years prior to screening, whilst FLAIR patients were treatment-naïve before being virologically suppressed using abacavir/dolutegravir/emtricitabine over 20 weeks. Non-inferiority in both trials was concluded if the upper limit of a two-sided 95% confidence interval evaluating the difference between LA and oral therapy in terms of percentage of participants with viral load (HIV RNA in the blood) of <50 copies/mL at 48 weeks, was less than six percentage points.

ART efficacy was measured by virologic response, i.e. viral suppression (HIV RNA <50 copies/mL), and immunological response (average increase in CD4 cell count). These values, as reported from the published pooled analysis of the ATLAS and FLAIR trials, are presented for CAB+RPV LA, combination ART (cART) and pooled across the two treatment arms, in Table 1.

**Table 1:** ART efficacy by virologic suppression and change in CD4 cell count.

Efficacy profile	Source	Virologic suppression at 48 weeks (%)*	Baseline CD4 count (cells/mm^3^)	48-week change in CD4 cell count
		Mean (SE)	Mean (SD)	Mean (SD)
Pooled efficacy	Pooled from ATLAS and FLAIR [27]	93.74 (0.70)	671.49 (268.99)	36.05 (189.37)
CAB+RPV LA		93.06 (1.05)	672.73 (264.48)	23.65 (194.64)
cART		94.42 (0.94)	670.25 (273.42)	47.82 (183.46)

Abbreviations: CAB: cabotegravir; cART: combination antiretroviral therapy; CD4: cluster of differentiation 4; LA: long-acting; RPV: rilpivirine; SD: standard deviation; SE: standard error.

*Viral suppression is defined by HIV RNA < 50 copies/mL blood

Table 2 shows the first, second and third year onwards figures for virologic and non-virologic discontinuation, based on the pooled trials.

**Table 2:** First, second and third year onwards figures for virologic and non-virologic discontinuation.

Efficacy profile	Source	Time point	Virologic discontinuation at 48 weeks (%)	Non-virologic discontinuation at 48 weeks (%)
			Mean (SE)	Mean (SE)
Pooled efficacy	Pooled from ATLAS and FLAIR [27]	Year 1	0.16 (0.12)	0.41 (0.19)
		Year 2	0.16 (0.12)	0.41 (0.19)
		Year 3+	0.16 (0.12)	0.41 (0.19)
CAB+RPV LA		Year 1	0.17 (0.17)	0.47 (0.28)
		Year 2	0.16 (0.12)	0.41 (0.19)
		Year 3+	0.16 (0.12)	0.41 (0.19)
cART		Year 1	0.15 (0.16)	0.36 (0.25)
		Year 2	0.16 (0.12)	0.41 (0.19)
		Year 3+	0.16 (0.12)	0.41 (0.19)

Abbreviations: CAB: cabotegravir; cART: combination antiretroviral therapy; LA: long-acting; RPV: rilpivirine; SE: standard error.

Adverse events (AEs), including injection site reactions (ISRs) are modelled via monthly, treatment-specific probabilities and are associated with a per-event cost and a monthly utility decrement. ADEs are modelled as a function of both CD4 cell count and time on treatment (S1 Appendix, Section 5).

### Adherence

Improved adherence is associated with an improved likelihood of viral suppression and a decreased likelihood of viral rebound and developing viral resistance to ART [9, 28, 29]. Adjustments to virologic suppression and rebound probabilities are based on Ross et al. [15] which reported the relationship between viral suppression at six months from ART initiation and a patient’s medication possession ratio. An equation was fitted to the observed data to calculate estimated viral suppression at different adherence levels (S1 Appendix, Section 2). Costs were adjusted for adherence: if a cohort of patients was 80% adherent, 80% of the regimen cost was assumed to be accrued, thus, no pill wastage was assumed.

Adherence to daily oral regimens in Canada overall, as well as in males, females and those with aboriginal ancestry were estimated from Samji et al. which reports data collected from eight cohorts situated in three Canadian provinces [30]. Reported values for the proportion of patients experiencing at least one treatment interruption, average follow-up and average time until treatment resumed were combined to estimate an 8.12% reduction in adherence to daily oral regimens in Canada (S1 Appendix, section 2). Similarly, reduction in adherence figures were derived for males (6.93%), females (13.25%) and those with aboriginal ancestry (19.48%).

CAB+RPV LA is formulated as a prolonged-release nanosuspension administered by IM injection and has a half-life of approximately 40 days, compared to 14 hours for oral DTG [20]. This allows two-monthly dosing with a flexibility window of seven days before and after the planned injection date. Because CAB+RPV LA is a DOT, HCPs control when doses are administered and can prescribe oral CAB + RPV between injections (oral bridging) in case an appointment is missed, or discontinue LA therapy for patients who cannot attend regular visits. This contrasts with daily oral regimens, where HCPs cannot observe their patients’ adherence, and is why LA DOT is modelled independently of adherence to daily dosing as in previous modelling studies [15], assuming the same efficacy and discontinuation as in combined Phase III trials. This assumption is further supported by the LATTE-2 study of CAB+RPV LA in which participants with a visit outside of the 14-day flexibility window experienced no virologic failure [19].

### Viral transmission

The cost-effectiveness model (CEM) was adapted to account for the impact of onwards HIV transmission, based on a targeted literature review. Patients are stratified into one of several risk groups (e.g. low-risk heterosexual or men who have sex with men [MSM]). The model estimates the number of onward infections, utilising the lifetime viral load health state occupancy of each risk group and predicting accrued costs, life years (LYs) and quality-adjusted life years (QALYs). It is assumed that the modelled cohort may only contribute to onwards HIV infections if they have a viral load ≥50 copies/mL, since a viral load below this (<50) is considered to be ‘undetectable’, therefore it is only during periods of increased viral load that patients may infect others. Subsequently, time spent in the higher viral load state is combined with the time-dependent risk of transmission (based on risk group-specific behaviour characteristics) to estimate the number of onwards HIV infections attributed to the initial cohort. Conservatively, only infections to the direct partners of the modelled cohort are considered within the model. The viral transmission module is described in detail in S2 Appendix.

### Utilities

Health-related quality of life is captured through utility values applied to each of the CD4 cell count health states and utility decrements applied based on the occurrence of ISRs and CVD (Table 3), derived from five open-label studies in patients treated with ART [31] (see S1 Appendix, Section 7 for details).

**Table 3:** Health related quality of life by CD4 health state.

CD4 cell count category (cells/mm^3^)	Mean	SE	Source
≥500	0.798	0.052	Kauf (2008) [31]
350–<500	0.784	0.059	
200–<350	0.778	0.053	
50–<200	0.750	0.058	
<50	0.742	0.058	
Utility decrements			
CVD: initial event	0.283	0.028	Ara (2009) [32]
CVD: chronic	0.156	0.016	
ISR	0.01	0.001*	ATLAS and FLAIR [27]

Abbreviations: CD4: cluster of differentiation 4; CVD: cardiovascular disease; ISR: injection site reaction; SE: standard error

*: SE assumed 10% of mean.

### Economic parameters

A discount rate of 1.5% was applied to both costs and health benefits as per Canadian Agency for Drugs and Technologies in Health (CADTH) guidance [33]. A Canadian health service cost perspective was adopted, with all costs inflated to 2017 prices using Canadian inflation indices [34]. The cost of SoC cART was set to a weighted average of ARTs comprising 95% of 2019 Canadian market share (CAD $1,232.76 per month). As the price of CAB+RPV LA is not yet available, this was set to a simple average of INSTI single tablet regimens ($1,227.00 per month). A cost of $2,515.52 per month was used for salvage therapy [35]. For CAB+RPV LA, administration costs of $35.15 for the oral initiation and first injectable cycles and $8.79 for all subsequent cycles were applied, corresponding to one hour and 15 minutes of a nurse’s time, respectively [36]. Additional details can be found in S1 Appendix, Section 9.

### Base case analysis

Utilising treatment-specific efficacy data pooled from the ATLAS and FLAIR trials [27], the cost-effectiveness of the every-two-month CAB+RPV LA (treatment with an initial loading dose followed by CAB LA 600 mg and RPV LA 900 mg every eight weeks) was evaluated in comparison to cART. The impact of removing suboptimal adherence to daily dosing was assessed both with and without accounting for the impact on subsequent onwards viral transmission. Incremental costs, QALYs and cases of HIV averted were estimated, per 1,000 patients treated as well as for the prevalent HIV-infected population of Canada.

### Deterministic sensitivity analysis

Key model parameters in the base case analysis, with and without the viral transmission component, were varied, with the impact on outputs displayed as tornado plots. This allowed the most influential individual parameters to be identified. Details of all parameters varied can be found in S1 Appendix, Section 10.

Given the extent of inter-dependence between the parameters of the viral transmission model but the lack of data to accurately quantify these correlations, probabilistic sensitivity analysis was deemed noninformative and so was not performed, in line with guidelines in this area [37].

### Scenario analysis: Treatment adherence in isolation

The scenario analysis assessed the impact of adherence (and subsequent onwards viral transmission) in isolation, by simulating two treatments identical in all other respects and varying adherence only. This was achieved by combining pooled efficacy data from ATLAS and FLAIR across the trial arms, resulting in identical efficacy profiles for both interventions. This analysis therefore assessed LA DOT more generally, rather than the specific case of CAB+RPV LA. This approach is supported by non-inferiority results from ATLAS and FLAIR, showing no significant differences between the LA DOT, CAB+RPV LA, and oral cART.

LA DOT was compared to current oral therapy regimens, given variations in oral treatment adherence. The impact on lifetime costs, QALYs, LYs and onwards infections was estimated.

### Ethics statement

As this article is based on previously conducted research and does not involve any new studies of human or animal subjects; ethics approval and consent to participate were not required.

## Results

### Base case analysis

**Fig 2:**
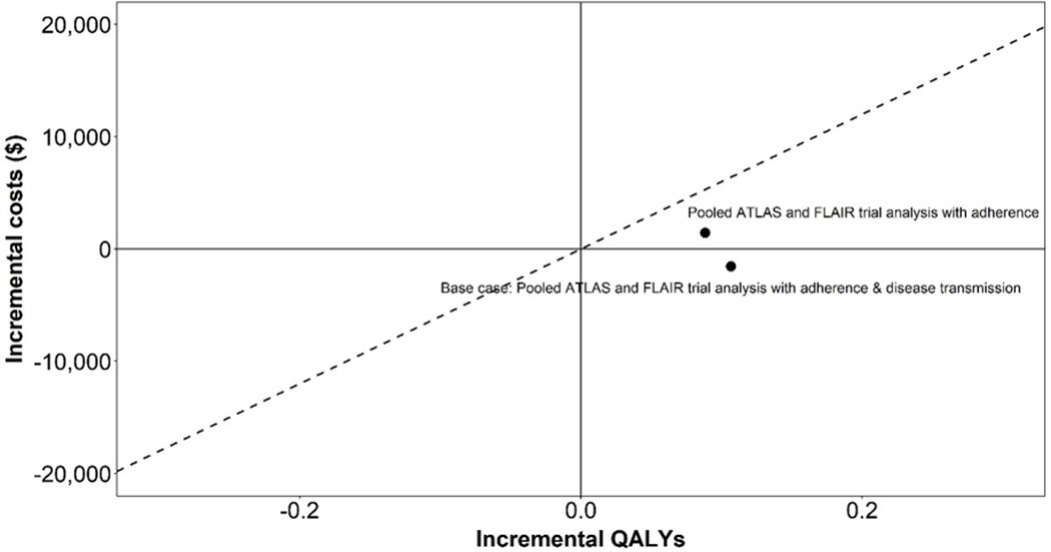
Cost-effectiveness plane (CAB+RPV LA vs oral cART). Abbreviations: QALY: quality adjusted life year.

In the analysis comparing the LA DOT CAB+RPV LA to cART, when including adherence and onwards viral transmission, CAB+RPV LA was predicted to dominate cART (Fig 2), resulting, per 1,000 patients treated, in lifetime cost-savings of $1.5 million, QALY and life-year gains of 107 and 138 respectively and 3 new HIV cases averted (Table 4).

**Table 4:** Cost-effectiveness results with and without impact of onwards viral transmission (CAB+RPV LA versus oral cART).

	Adherence cost-effectiveness analysis			Adherence and onwards viral transmission cost-effectiveness analysis		
	CAB+RPV LA	Oral cART	Incremental	CAB+RPV LA	Oral cART	Incremental
Treated cohort						
Total LYs	23.852	23.736	0.116	23.852	23.736	0.116
Total QALYs	17.332	17.243	0.088	17.332	17.243	0.088
Total costs ($; CAD)	621,393	619,974	1,420	621,393	619,974	1,420
Viral transmission						
LYs lost due to viral transmission	NA	NA	NA	0.143	0.165	0.022*
QALYs lost due to viral transmission	NA	NA	NA	0.118	0.136	0.018*
Cost of viral transmission ($; CAD)	NA	NA	NA	19,133	22,103	-2,969
Cost-effectiveness ($; CAD)						
Cost / LY	12,287			CAB+RPV LA dominant		
Cost / QALY	16,062			CAB+RPV LA dominant		

Abbreviations: CAB: cabotegravir; CAD: Canadian dollar; cART: combination antiretroviral therapy; CE: cost-effective; LA: long-acting; LY: life year; NA: not applicable; QALY: quality-adjusted life year; RPV: rilpivirine

*Fewer LYs/QALYs lost is presented as a gain to aid interpretation

QALYs and LYs rounded to 3 decimal places

QALYs, LYs and costs are per-patient.

### Impact of treatment adherence

Patients with suboptimal adherence to daily dosing are less likely to achieve viral suppression and thus more likely to discontinue virologically, progressing more rapidly towards salvage therapies associated with reduced CD4 and increased costs. Patients who are not virologically suppressed are also at increased risk of transmitting HIV to others, with a corresponding impact on QALYs and costs resulting from new HIV cases. The majority of the QALY gain realised by CAB+RPV LA per patient (0.106) is attributable to the direct consequences of improved adherence on the modelled cohort (0.088), with a smaller gain attributable to the reduction in HIV transmissions (0.018) (Table 5).

**Table 5:** Benefits of LA DOT extrapolated to the HIV-infected population of Canada.

Population	Number of patients	Future transmission avoided	QALYs gained	LYs gained	Cost savings ($; CAD)
Canadian HIV Prevalence (2016)					
Total	63,110	201	7816	9917	211,038,174
Indigenous population	6,055	54	1847	2319	21,113,702
Females	14,520	81	2849	3551	70,529,640
Males	48,590	130	5177	6605	139,978,013

Abbreviations: CAD: Canadian dollar; IDU: injection drug use; LY: life year; QALY: quality-adjusted life year.

### Deterministic sensitivity analysis

**Fig 3:**
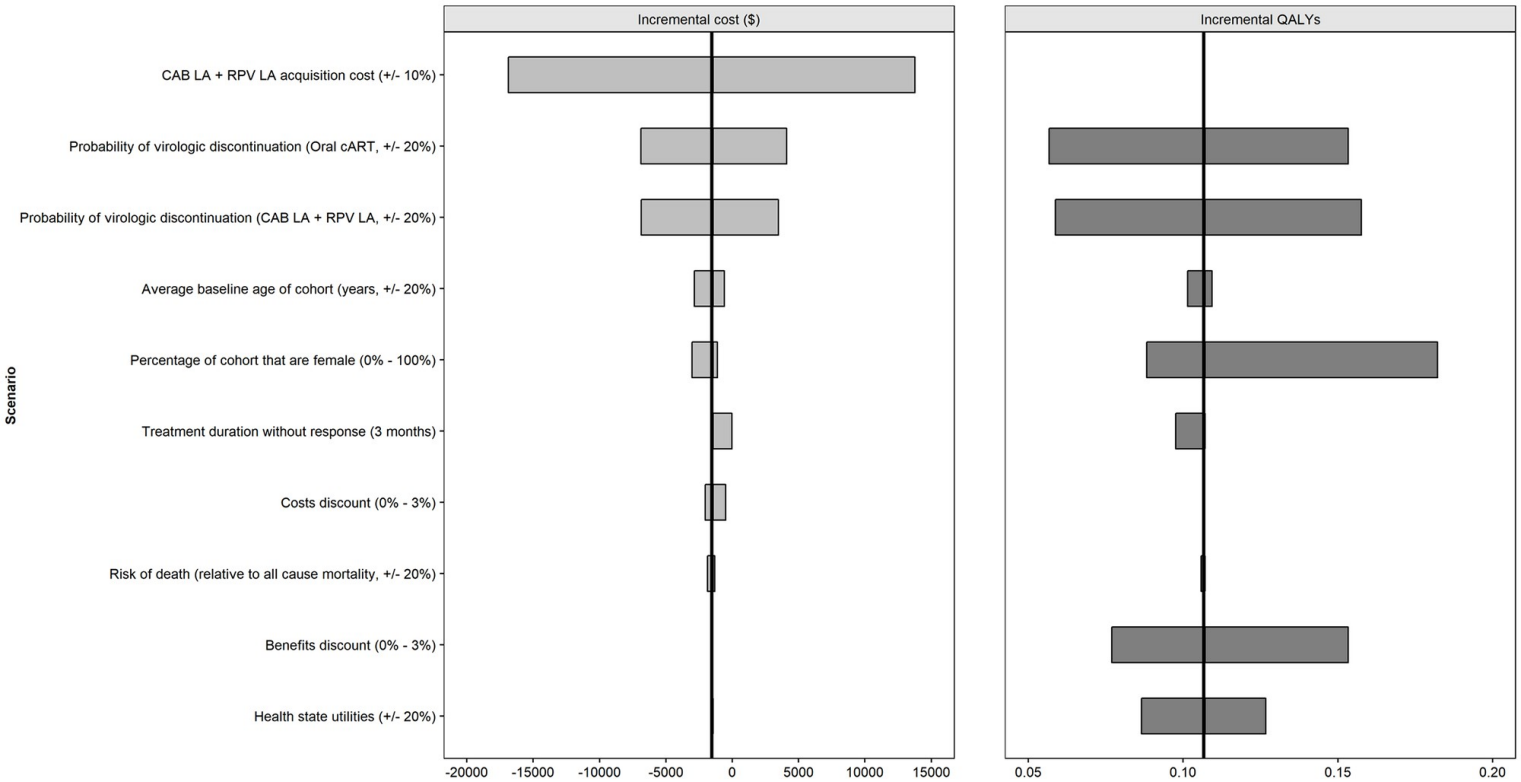
Deterministic (one-way) sensitivity analysis (CAB+RPV LA vs oral cART) CEM parameters. Abbreviations: CAB: cabotegravir; cART: combination antiretroviral therapy; LA: long-acting; QALY: quality adjusted life year; RPV: rilpivirine.

The results of the deterministic sensitivity analysis for the CEM are shown in Fig 3.

Changes in the probability of virologic discontinuation, the acquisition cost of CAB+RPV LA, the benefit discount rate and the proportion of females had the greatest impact on outcomes.

**Fig 4:**
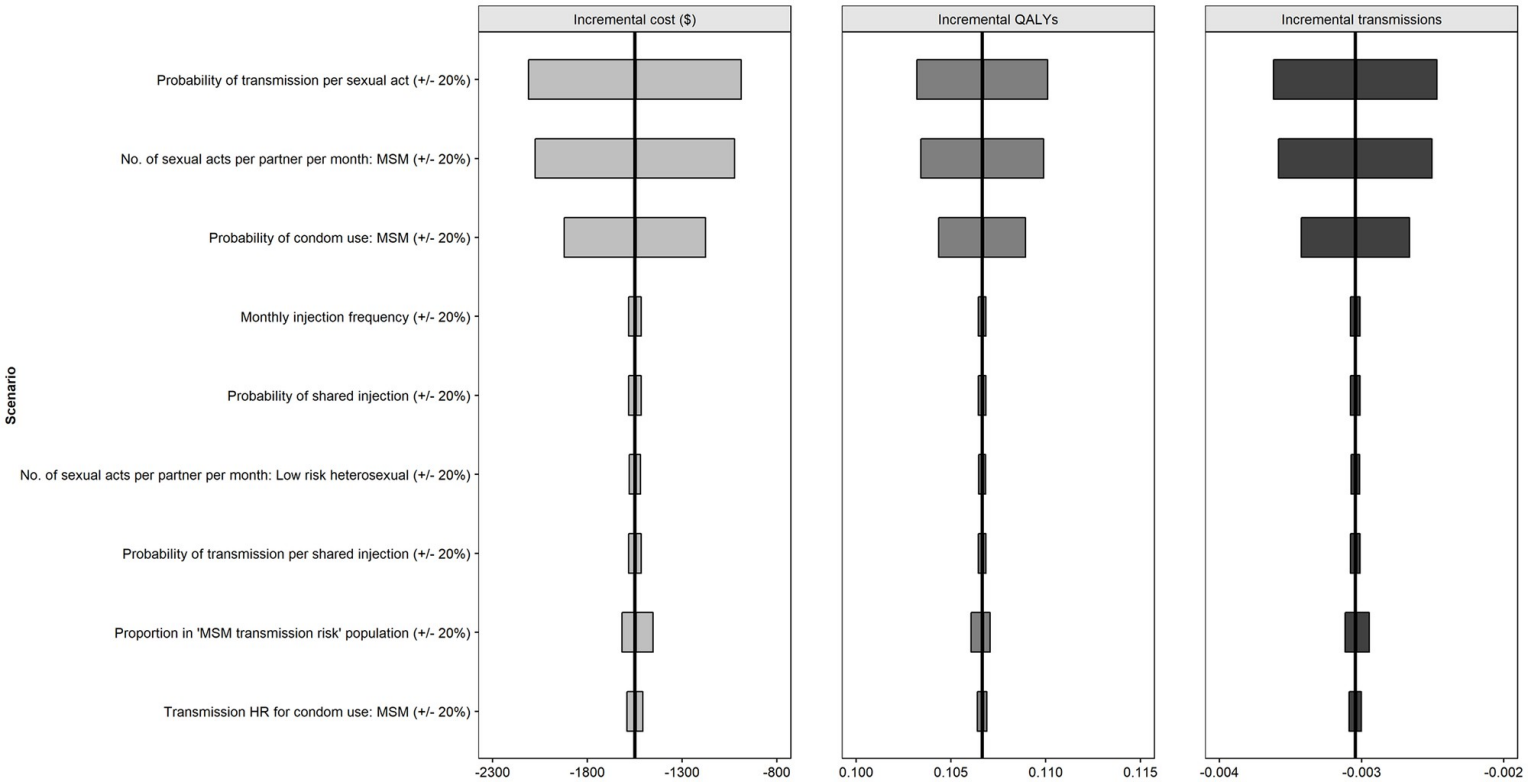
Deterministic (one-way) sensitivity analysis (CAB+RPV LA vs oral cART) viral transmission parameters. Abbreviations: HR: hazard ratio; MSM: men who have sex with men; QALY: quality adjusted life year.

For the viral transmission model (Fig 4), the probability of transmission per sexual act and both the probability of condom use and the number of sexual acts per partner per month in the MSM population had the greatest impact on the results.

### Scenario analysis: Treatment adherence in isolation

**Fig 5:**
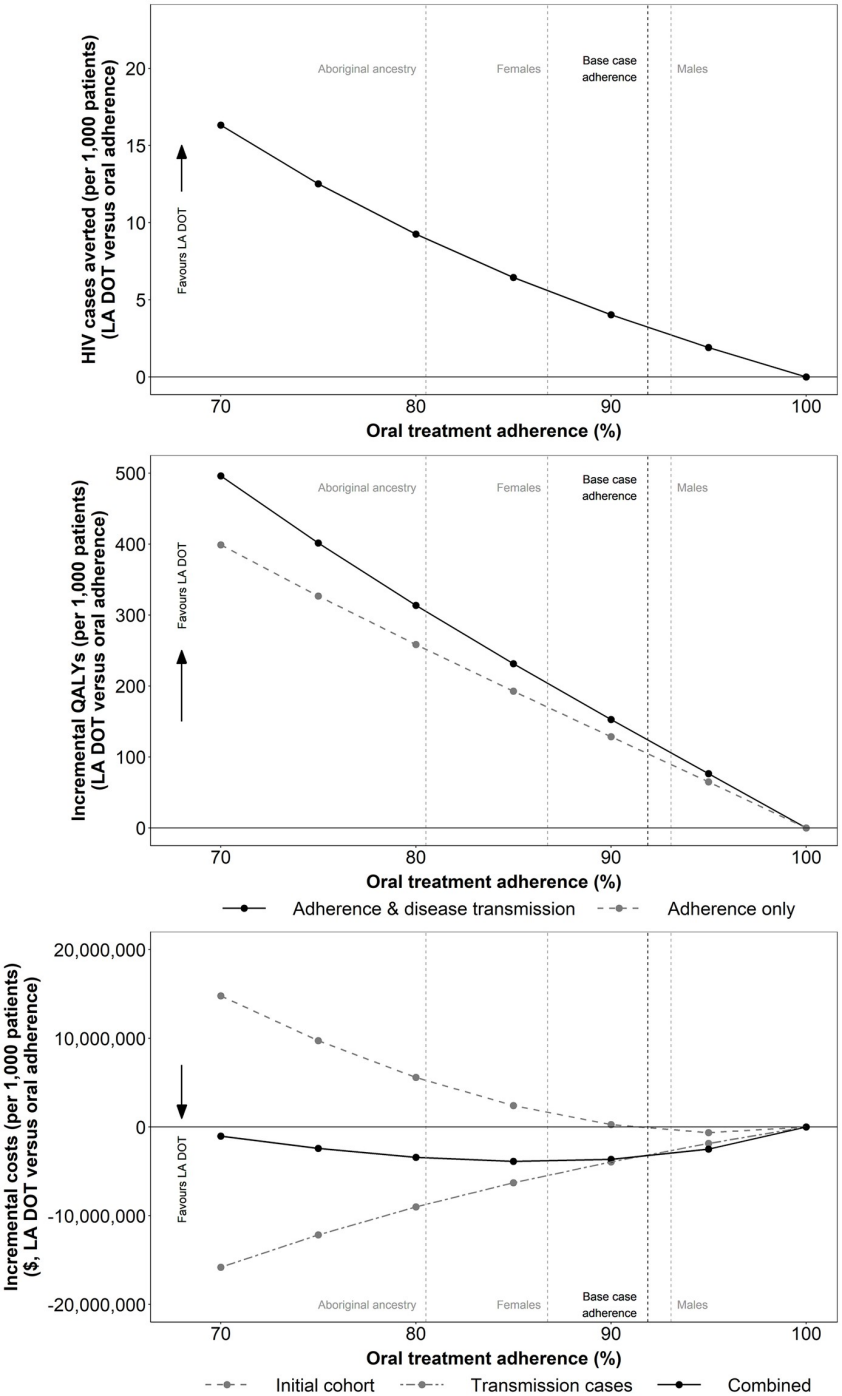
Impact of average baseline population treatment adherence on the incremental number of transmissions, QALYs and total costs per 1,000 patients treated (LA DOT vs oral adherence). Abbreviations: DOT: directly observed therapy; LA: long-acting; QALY: quality adjusted life year.

The impact of eliminating suboptimal adherence in the daily oral arm is presented in Fig 5.

As average baseline treatment adherence in the comparator arm decreases, the estimated benefits of improving adherence to optimal levels are increased, with fewer onwards infections and greater QALY and LY gains. Assuming a mean baseline treatment adherence reduction of 8.12% in Canada [30], improving adherence to optimal levels was predicted to avert three HIV transmissions and to generate cost savings of $3.3 million as well as QALY and LY gains of 124 and 157, respectively, per 1,000 patients treated.

The greatest benefits were observed when assessing the impact of improved adherence in higher risk populations with lower average baseline adherence levels [30]. In female patients, for example, average baseline adherence reduction was estimated at 13.25%, and consequently, improving adherence was associated with cost savings of $4.9 million and QALY and LY gains of 196 and 245, respectively, per 1,000 patients treated.

Extrapolating outcomes to a population level (Table 5), assuming mean Canadian levels of baseline adherence [30], highlights the significant benefit associated with improving treatment adherence. Improving adherence to optimal levels in the prevalent Canadian HIV population (2016 estimate), is predicted to result in QALY gains of 7,816, realised in part through the avoidance of 201 direct onwards infections over the lifetime of the prevalent cohort.

## Discussion

HIV treatment requires adherence to daily oral ART to maintain viral suppression over time. Achieving the 95-95-95 UNAIDS target may be impeded by the difficulties inherent in improving treatment adherence due to the complex range of causes underlying non-adherence [8, 13, 14]. CAB+RPV LA, the first complete LA ART regimen, demonstrated noninferior efficacy against 3-drug regimens. Participants in clinical trials reported significantly greater treatment satisfaction with and preference for CAB+RPV LA compared to daily oral SoC [21, 22]. Providing patients with new preferred treatment options may improve the proportion of PLWH receiving and adhering to treatment, contributing to UNAIDS 95-95-95 targets to eradicate HIV. LA DOT removes the need for adherence to daily dosing, which is assumed to result in improved adherence over the long term, in turn leading to improved viral suppression and a lower rate of onwards viral transmissions compared to SoC. This results in CAB+RPV LA being a dominant intervention, delivering increased health benefits alongside cost savings.

Improving adherence to HIV therapies requiring daily oral dosing, particularly in vulnerable patients, may be difficult without measures such as resource intensive outreach programmes [38]. The societal costs of daily DOT ART and intensive adherence case management (IACM; self-administered ART with visits every 1–2 weeks to overcome barriers to adherence) have been compared to SoC, with IACM shown to generate cost savings of $3775 per participant [39]. CAB+RPV LA has the potential to dominate the combination of ART regimens with adherence-enhancing interventions because CAB+RPV LA reduces the frequency and burden of medication and provides uninterrupted efficacy. The value of such improvement is demonstrated herein for the every-two-month CAB+RPV LA compared to oral cART.

Economic evaluations comparing two daily oral regimens can use trial-based analyses which do not consider adherence nor transmission since no difference is expected. However, as demonstrated by others [15], accurately assessing new ART regimens with different modes of administration requires the inclusion of adherence in the model structure.

The present study contributes to this literature by incorporating the impact of treatment adherence on onwards viral transmission and utilising clinical data for the recently developed CAB+RPV LA. The importance of modelling the impact on subsequent viral transmission when evaluating new HIV treatments with different modes of administration is clearly demonstrated, leading to an increased gain in QALYs and a reduction in costs, sufficient to cause CAB+RPV LA to go from being cost-effective to being the dominant intervention (Fig 2).

The study has a number of limitations. The results are based on the synthesis of evidence from multiple sources and are therefore subject to a degree of uncertainty. In addition, certain assumptions were required. Our analysis assumes that PLWH outside of clinical trials would self-select to receive IM injections in the same way as trial participants, and thus would exhibit optimal adherence. Additionally, if an injection was missed, the 14-day flexibility window would ensure patients remained virally suppressed, provided they obtained an appointment within this time [19]. However, in reality, some patients might not adhere adequately to the visit schedule. It is likely that such patients would not continue to be prescribed CAB LA + RPV LA. Secondly, the viral transmission model captures only direct transmissions, providing an inherent underestimate of the actual transmissions avoided. A dynamic transmission model would further emphasise the potential for LA DOT to reduce the transmission of HIV. Further research into the impact of novel HIV treatments on adherence and onwards viral transmission is therefore warranted, and a renewed focus in HIV drug development on factors with the potential to improve treatment adherence may be needed. Lastly, the model does not capture potential utility improvements associated with the advantages of LA therapy, such as freedom from concerns related to scheduling daily treatment around lifestyle factors, removal of the psychological burden of daily treatment and the associated reminder of one’s HIV status, and removal of fears over unwanted disclosure of HIV status resulting from discovery of the patient’s pills by others.

## Conclusions

This study clearly demonstrates the impact of improved adherence on health economic outcomes and incorporates the additional benefit of reduced onwards viral transmissions, using trial data for CAB+RPV LA. The introduction of long-acting therapies such as CAB+RPV LA, which offers less frequent, directly observed dosing, has the potential to aid progress towards meeting the UNAIDS 95-95-95 target.

## Supporting information

S1 Appendix Additional details for the cost-effectiveness model. (DOCX)

S2 Appendix Additional details relating to the onwards viral transmission module of the cost-effectiveness model. (DOCX)
