# Supplementary material for: Cost-effectiveness of the long-acting regimen cabotegravir plus rilpivirine for the treatment of HIV-1 and its potential impact on adherence and viral transmission: A modelling study
Source: PLoS One. 2021 Feb 2;16(2):e0245955. doi: 10.1371/journal.pone.0245955 (PMC7853524; doi:10.1371/journal.pone.0245955)
Supplement: S1 Appendix — (DOCX) [file pone.0245955.s001.docx]

**S1 Appendix**

1. **Model** structure

The developed model is a deterministic hybrid Markov state transition model. Patients with HIV are at risk of experiencing treatment failure (predominantly due to resistance development, failure to achieve virologic suppression or adverse events), resulting in discontinuation of current therapy. To capture this complexity, a traditional Markov process was combined with a decision tree process, which manages treatment allocation and aggregates results across treatment lines. An internal decision process is also employed to manage patients exhibiting virologic failure and those discontinuing due to adverse events (AEs).

Schematics depicting the treatment pathways modelled and the within treatment line health states are presented in Fig 1.


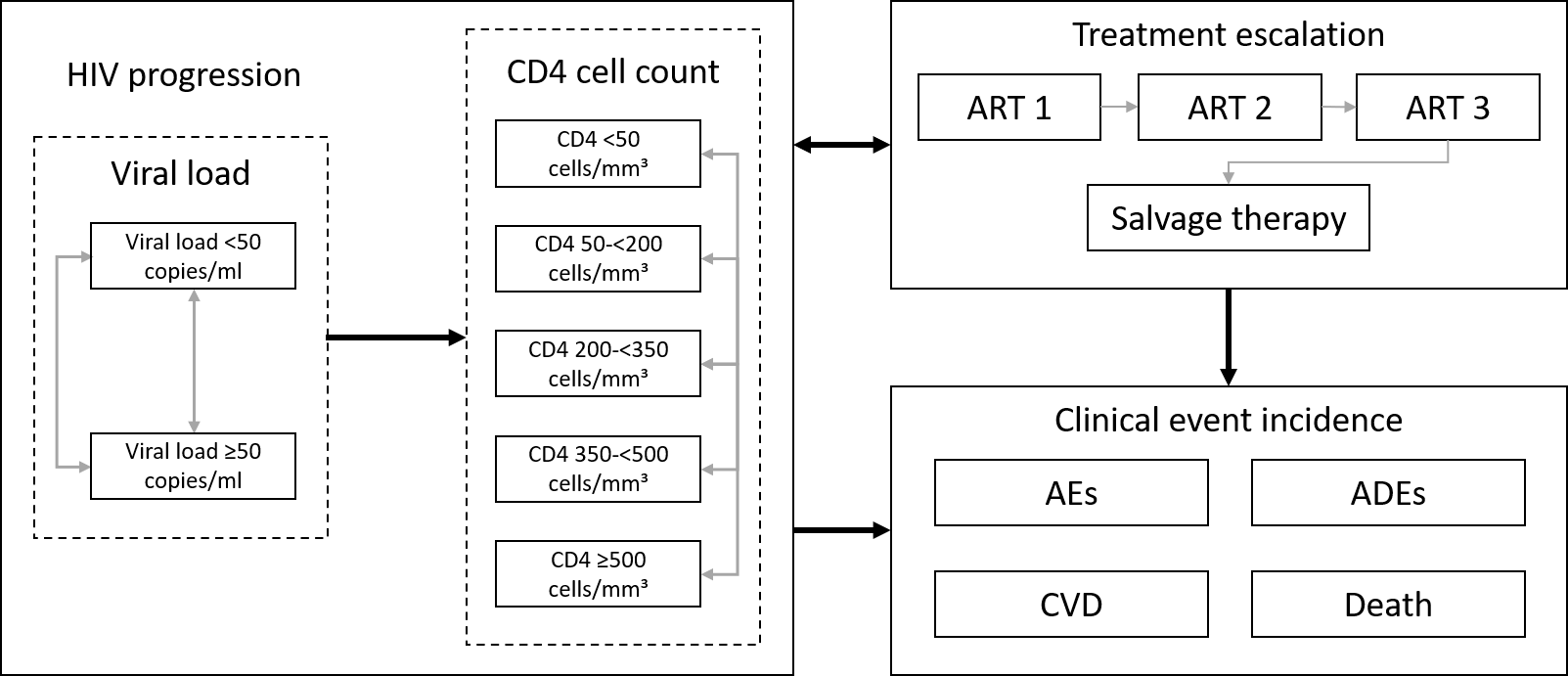


S1 Fig 1. Treatment pathway and within therapy health states

Health states included in the model are based upon treatment line, virologic response and cluster of differentiation 4 (CD4) cell count, with death as an absorbing state. Whilst not defined as explicit health states, patients are also subject to the risk of AIDS defining events (ADEs), treatment-related AEs and cardiovascular disease (CVD).

Upon model initiation, patients begin in the first antiretroviral therapy (ART) line (1^st^ Modelled Line). For each modelled arm, following discontinuation from the initial ART line patients are stratified amongst two possible subsequent ART regimens, decided by their reason for discontinuation (‘Non-virologic’ and ‘Virologic’). Following discontinuation from the first subsequent therapy, a similar stratification occurs. It is assumed that patients who discontinue due to ‘Virologic’ reasons develop resistance, and as such have poorer suppression rates in subsequent lines of therapy. To account for differing levels of resistance at entry to the ‘Salvage’ therapy line, patients may receive one of three ‘Salvage’ therapy efficacy profiles depending on the nature of their previous reasons for discontinuations.

Upon initiation into a given treatment line, patients enter the Markov process. Consistent with previous economic models[1, 2], health states included are based on viral load (<50 copies/mL, ≥50 copies/mL) and CD4 cell count (≥500 cells/mm^3^, 350–<500 cells/mm^3^, 200–<350 cells/mm^3^, 50–<200 cells/mm^3^, <50 cells/mm^3^) and death. During each cycle (one month), patients’ viral status may improve, decline or remain constant. Death is an absorbing state. In general, patients within each health state are assumed to be homogenous, except those who experience virologic failure.

In any of the first three modelled treatment lines, patients may discontinue treatment due to virologic failure or other non-virologic reasons. Patients receiving treatment within the Salvage therapy line are assumed to remain there for the remainder of the modelled horizon, with the Salvage therapy line acting as an absorbing health state with respect to treatment options and assumed to contain all potential therapies post-3^rd^ line treatment.

Discontinuation due to virologic failure is managed by the internal decision process:

- **Virologic failure:** so that a cohort does not indefinitely remain on a failing treatment regimen, an internal memory process identifies those who have occupied the high viral load state (≥50 copies/mL) for 1 month. Patients who have failed to achieve virologic suppression within this period discontinue their current therapy. Patients discontinuing due to virologic failure remain in the same CD4 health state in the subsequent treatment arm, whilst also remaining in the high viral load state.

Discontinuation due to viral rebound and other non-virologic reasons is managed through treatment-specific transition matrices:

- **Virologic rebound:** patients who are achieving virologic suppression (viral load <50 copies/mL) face a monthly probability of experiencing virologic rebound. Patients experiencing virologic rebound are assigned to the highest (viral load ≥50 copies/mL) viral load state in a subsequent treatment line.
- **Non-virologic reasons**: patients in any CD4 or viral load health state face a monthly probability of discontinuing their current line of therapy due to other non-virologic reasons. Patients who discontinue through this process remain in their existing CD4 and viral load health state.

A graphical representation of the treatment switching processes in presented in Fig 2.


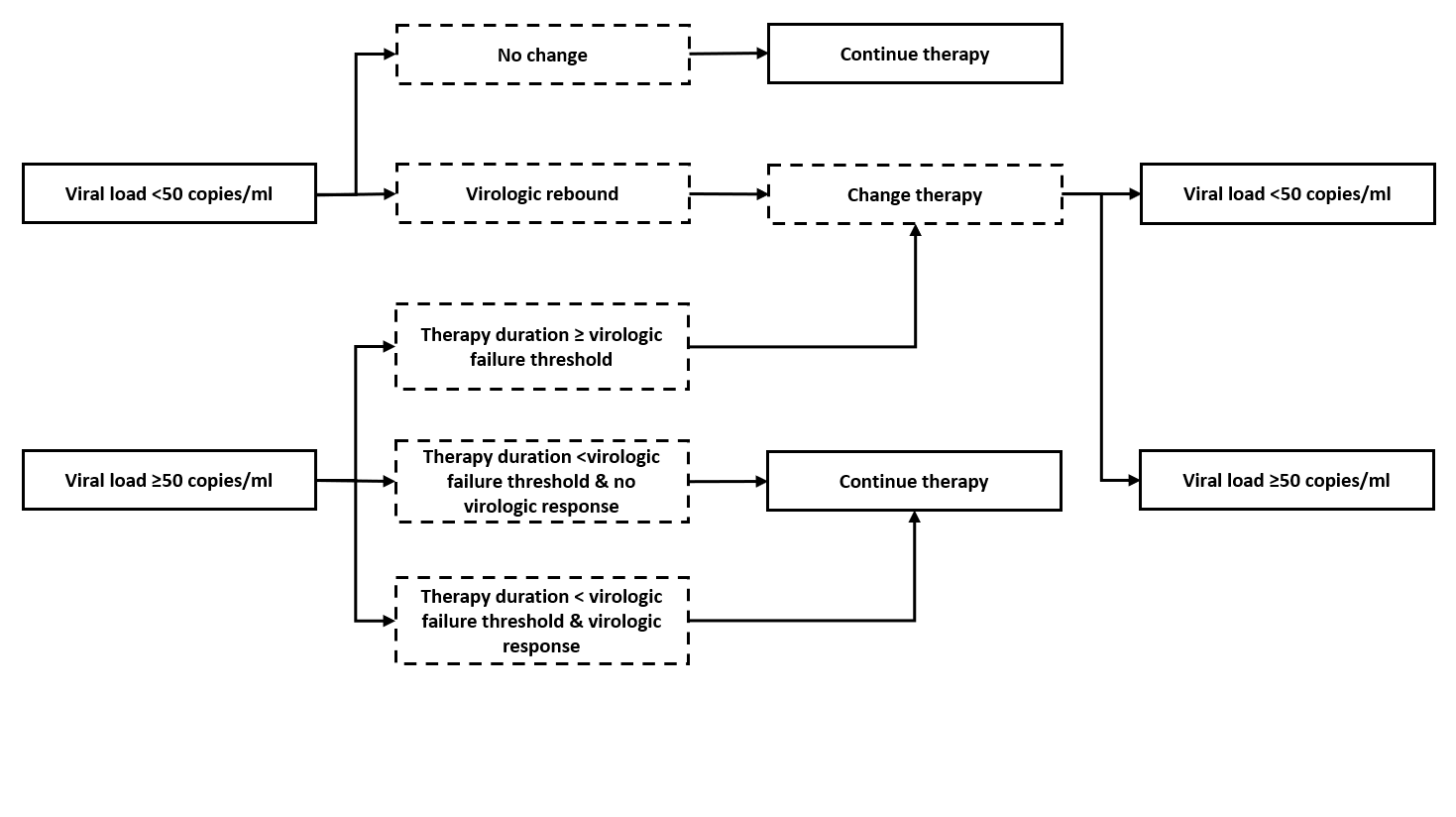


S1 Fig 2. Treatment switching decision processes

Where treatment switching is necessary, the decision tree allocates patients to the appropriate subsequent treatment. Once analyses have been completed for all treatment permutations, the decision tree aggregates results to inform the overall cohort results, as presented in Fig 3.


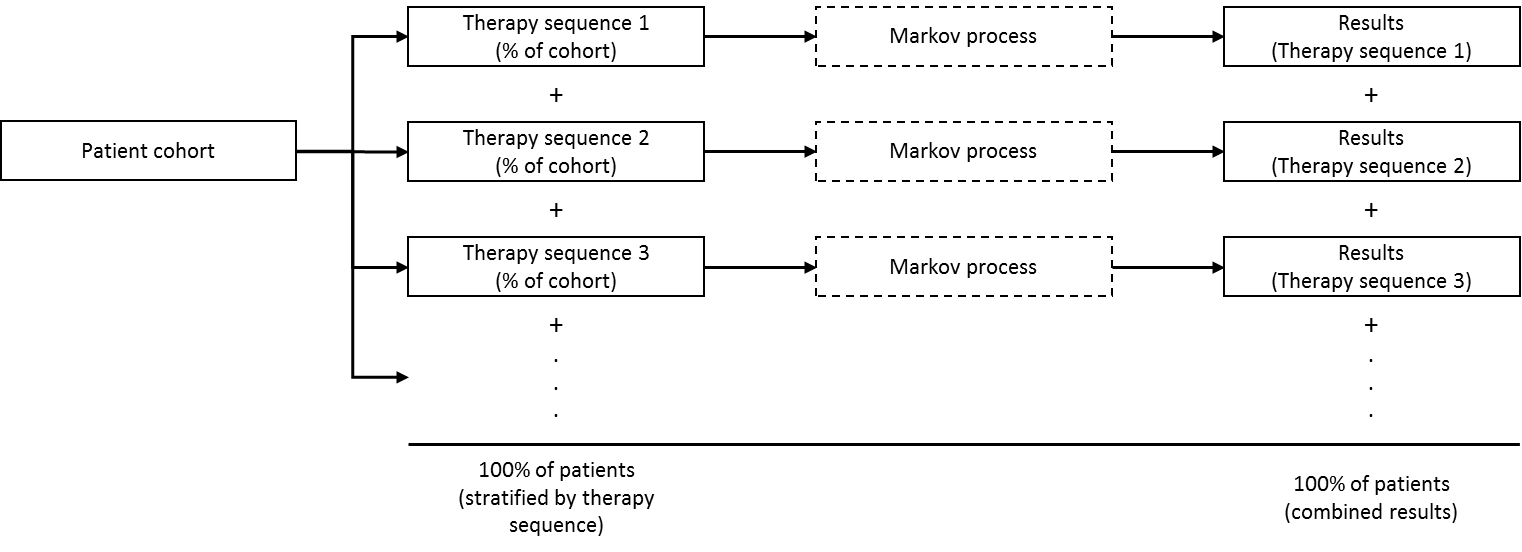


S1 Fig 3. Decision tree process

All patients receive three lines of ART, followed by one line of salvage treatment, depending on their treatment pathway and reasons for discontinuation.

S1 Table 1. Efficacy profile options

| First-line options | Second and third line options |
| --- | --- |
| Pooled comparator | Treatment experienced: stable switch[3] |
| CAB+RPV LA Q4W (derived from CAB+RPV LA pooled ATLAS and FLAIR trial data [4]) | Treatment experienced: failing switch[5] |
| cART (derived from pooling the comparator arms of FLAIR and ATLAS trials [4]) |  |
| Abbreviations: CAB: cabotegravir; cART: combination antiretroviral therapy; LA: long-acting; Q4W: administered once per 4 weeks; RPV: rilpivirine. | |

Efficacy profiles are chosen based on the reason for discontinuation, where virologic failure is considered to result in the development of antiviral resistance and poorer outcomes. Stable switch indicates a patient has discontinued due to reasons unrelated to the development of antiviral resistance and as such are expected to have better outcomes.

1. Adherence

The default reduction in adherence for oral therapies is derived from a study by Samji et al.[6]. The study reports the proportion of patients that have at least one treatment interruption (Prop TI, calculated using 1,860/7,633 i.e. 24.37%) over an average reported follow-up of 2.4 years (FU) and estimates that resumption of treatment occurs after 0.8 years (TR) (9.6 months, as reported by Samji et al.) on average. Adherence was subsequently calculated using the following calculation:

$$Reduction in adherence (8.12\%)= Prop TI \cdot\left( \frac{TR}{FU} \right)$$

$$8.12\%= 0.2437 \cdot\left( \frac{0.8}{2.4} \right)$$

Within the model, treatment with CAB+RPV LA is assumed to be associated with optimal adherence, due to the requirement for a medical practitioner to administer the injections.

Reduced adherence is associated with a reduction in the likelihood of viral suppression and an increased likelihood of patients experiencing viral rebound and developing resistance to ART. Adjustments to viral suppression are based on a study by Ross et al.[7]. The study reports the relationship between viral suppression at six months from ART initiation and medication possession ratio (MPR). The following equation was fitted to the observed data to calculate the estimated viral suppression:

$$Viral suppression=1.01111\cdot Adherence-0.05056$$

Subsequently, this equation is used to estimate viral suppression assuming 100% adherence and assuming a defined level of adherence, with the proportional difference between the two outcomes used to inflate or deflate virologic suppression input data. For example, in a cohort exhibiting 80% adherence, the above equation will produce a viral suppression estimate of 75.83%, this is then divided by the estimate for a fully adherent cohort, in this case 96.06% giving an adjustment factor of 0.79. Probability of viral suppression would be scaled down by multiplying by this factor, and probability of viral rebound will be scaled up through assuming that all patients that would have otherwise achieved viral suppression now experience viral rebound.

Oral regimen costs are also adjusted for adherence. For example, if a cohort of patients is 80% adherent in a given month, it is assumed they accrue only 80% of the regimen cost during that month (i.e. the model assumes no wastage).

1. Viral transmission

The onwards transmission of HIV is dependent on several factors, predominantly the likelihood of behaviour conducive to viral transmission (e.g. unsafe sexual activity or injection drug use) and the viral load history of patients. The viral transmission module is described in detail in S2 Appendix.

1. Model inputs
   1. Baseline characteristics

- **Mean age (years)**: influences rates of all-cause mortality and the risk of CVD
- **Percentage female (%)**: influences rates of all-cause mortality and the risk of CVD
- **Currently treated for hypertension (1/0)**: influences the risk of CVD (see Appendix A)
- **Percentage current smokers (%)**: influences the risk of CVD
- **Mean total cholesterol (mg/dL)**: influences the risk of CVD
- **Mean high-density lipoprotein cholesterol (mg/dL)**: influences the risk of CVD
- **Mean systolic blood pressure (mmHg)**: influences the risk of CVD
- **Baseline viral status**: relates to the percentage of patients in each CD4^+^ cell count category (≥500 cells/mm^3^, 350–<500 cells/mm^3^, 200–<350 cells/mm^3^, 50–<200 cells/mm^3^, <50 cells/mm^3^) for each viral load state (<50 copies/mL, ≥50 copies/mL)
- **Comorbidities (history of diabetes)**: influences the risk of CVD and eligibility for certain treatments

Model default baseline demographics can be found in S1 Table 2.

S1 Table 2. Baseline cohort profile

| **Variable** | **Input** | | |
| --- | --- | --- | --- |
|  | **Mean Value** | **SE** | **Source** |
| Age of cohort (years) | 39.35 | 0.33 | Pooled efficacy data |
| Percentage of cohort that are female (%) | 27.92% | 1.30% | Pooled efficacy data |
| Cohort treated for SBP (1/0) | 0 | 0 | Assumption |
| Current smokers (%) | 34.23% | 1.38% | FLAIR & ATLAS [4] |
| Total cholesterol (mg/dL) | 171.31 | 0.13 | FLAIR [4] |
| HDL cholesterol (mg/dL) | 49.32 | 0.02 | FLAIR [4] |
| SBP (mmHg) | 121.70 | 0.55 | FLAIR [4] |
| Percentage of cohort with a history of diabetes (%) | 1.94% | 0.58% | FLAIR [4] |
| Abbreviations: HDL: high-density lipoprotein cholesterol; SBP: systolic blood pressure; SE: standard error. | | | |

The default baseline viral status included in the model is presented in Table 3.

S1Table 3. Baseline viral status

| **Viral load (copies/mL)** | **CD4 cell count category (cells/mm^3^)** | **ATLAS** | | | **FLAIR** | | | **Pooled (ATLAS and FLAIR)** | | |
| --- | --- | --- | --- | --- | --- | --- | --- | --- | --- | --- |
|  |  | **Mean** | **SE** | **Source** | **Mean** | **SE** | **Source** | **Mean** | **SE** | **Source** |
| <50 | ≥500 | 73.54% | 1.78% | ATLAS [4] | 69.96% | 1.93% | FLAIR [4] | 71.83% | 1.31% | Pooled efficacy data |
|  | 350–<500 | 18.34% | 1.56% | ATLAS [4] | 21.91% | 1.74% | FLAIR [4] | 20.05% | 1.16% | Pooled efficacy data |
|  | 200–<350 | 8.12%* | 1.10% | ATLAS [4] | 8.13% | 1.15% | FLAIR [4] | 8.12% | 0.79% | Pooled efficacy data |
|  | 50–<200 | 0.00% | 0.00% | Assumed | 0.00% | 0.00% | FLAIR [4] | 0.00% | 0.00% | Pooled efficacy data |
|  | <50 | 0.00% | 0.00% | Assumed | 0.00% | 0.00% | FLAIR [4] | 0.00% | 0.00% | Pooled efficacy data |
| ≥50 | ≥500 | 0.00% | 0.00% | Assumed | 0.00% | 0.00% | FLAIR [4] | 0.00% | 0.00% | Pooled efficacy data |
|  | 350–<500 | 0.00% | 0.00% | Assumed | 0.00% | 0.00% | FLAIR [4] | 0.00% | 0.00% | Pooled efficacy data |
|  | 200–<350 | 0.00% | 0.00% | Assumed | 0.00% | 0.00% | FLAIR [4] | 0.00% | 0.00% | Pooled efficacy data |
|  | 50–<200 | 0.00% | 0.00% | Assumed | 0.00% | 0.00% | FLAIR [4] | 0.00% | 0.00% | Pooled efficacy data |
|  | <50 | 0.00% | 0.00% | Assumed | 0.00% | 0.00% | FLAIR [4] | 0.00% | 0.00% | Pooled efficacy data |
| Abbreviations: CD4: cluster of differentiation 4; SE: standard error  *Assumed that all patients with CD4 <350 (as defined in ATLAS [4]) fall within this category. | | | | | | | | | | |

1. HIV-related clinical inputs

In each cycle (one month), patients are at risk of experiencing an ADE. The probability associated with specific ADEs are a factor of both CD4 cell count and time since model initiation (time on treatment). The incidence of ADEs influences mortality, disease management costs and quality of life.

The five ADEs modelled are the following classes of opportunistic infection (OI):

- Acute viral
- Acute bacterial
- Acute fungal
- Acute protozoan
- Other

Probabilities for each ADE can be found in Table 4. The source publication describing ADE probabilities over time observed that in some cases, risk of ADE increased with increasing CD4 cell count, in order to better replicate known viral progression, the lowest probability by CD4 cell count was carried forward, so that improved CD4 cell count does not yield a higher likelihood of ADEs.

S1 Table 4. AIDS-defining event incidence

| **Time on treatment** | **Opportunistic infection** | **Probability of experiencing an ADE (mean value)** | | | | | **Source** |
| --- | --- | --- | --- | --- | --- | --- | --- |
|  |  | **CD4 <50** | **CD4 50-<200** | **CD4 200-<350** | **CD4 350-<500** | **CD4 ≥500** |  |
| 0-6 months | Acute viral OI  Acute bacterial OI  Acute fungal OI  Acute protozoal OI  Other OI | 0.0071  0.0070  0.0049  0.0021  0.0036 | 0.0033  0.0022  0.0022  0.0006  0.0020 | 0.0008  0.0006  0.0003  0.0002  0.0000 | 0.0008  0.0004  0.0001  0.0001  0.0000 | 0.0008  0.0004  0.0001  0.0001  0.0000 | ARAMIS technical report (unpublished data)  Lowest value for each time-point by CD4 cell count carried forward |
| 7-12 months | Acute viral OI  Acute bacterial OI  Acute fungal OI  Acute protozoal OI  Other OI | 0.0039  0.0027  0.0018  0.0018  0.0022 | 0.0010  0.0009  0.0013  0.0004  0.0014 | 0.0003  0.0001  0.0002  0.0001  0.0007 | 0.0003  0.0001  0.0002  0.0001  0.0003 | 0.0002  0.0001  0.0001  0.0001  0.0003 |  |
| 13-24 months | Acute viral OI  Acute bacterial OI  Acute fungal OI  Acute protozoal OI  Other OI | 0.0019  0.0022  0.0016  0.0015  0.0014 | 0.0005  0.0008  0.0011  0.0004  0.0009 | 0.0002  0.0001  0.0002  0.0001  0.0004 | 0.0002  0.0001  0.0002  0.0001  0.0002 | 0.0001  0.0001  0.0001  0.0001  0.0002 |  |
| 25-36 months | Acute viral OI  Acute bacterial OI  Acute fungal OI  Acute protozoal OI  Other OI | 0.0005  0.0012  0.0015  0.0008  0.0009 | 0.0001  0.0004  0.0011  0.0002  0.0006 | 0.0000  0.0000  0.0001  0.0000  0.0003 | 0.0000  0.0000  0.0001  0.0000  0.0001 | 0.0000  0.0000  0.0001  0.0000  0.0001 |  |
| 36 months+ | Acute viral OI  Acute bacterial OI  Acute fungal OI  Acute protozoal OI  Other OI | 0.0005  0.0012  0.0015  0.0008  0.0009 | 0.0001  0.0004  0.0011  0.0002  0.0006 | 0.0000  0.0000  0.0001  0.0000  0.0003 | 0.0000  0.0000  0.0001  0.0000  0.0001 | 0.0000  0.0000  0.0001  0.0000  0.0001 |  |
| Abbreviations: ADE: AIDS-defining event; CD4: cluster of differentiation 4; OI: opportunistic infection  SE assumed to be 10% of mean for all inputs. | | | | | | | |

- 1. Cardiovascular disease

Within each cycle, patients are at risk of developing CVD. The probability of developing CVD is derived from the baseline cohort characteristics (age, total cholesterol, high-density lipid profile, blood pressure, smoking and diabetes status) using the Framingham risk equation (see Appendix A.)[8].

This probability is updated annually to reflect ageing of the cohort. All other inputs utilised in the Framingham risk equation are assumed to remain constant. Patients who develop CVD have it for the remainder of their lifetime. CVD influences mortality, disease management costs and quality of life.

1. Treatment-related clinical inputs

Treatment-related effects are captured according to the following:

- Efficacy:
  - Virologic response, defined as the achievement of HIV RNA <50 copies/ml
  - Immunological response, defined as the average increase in CD4 cell count
- Safety:
  - AEs (up to 20 grade 3 and 4 AEs)
  - Discontinuation
- Lipid profiles:
  - Total cholesterol change
  - High-density lipoprotein (HDL) cholesterol change
  1. Efficacy

ART efficacy is measured by virologic response, defined as the achievement of a viral load (HIV RNA) <50 copies/mL, and immunological response, defined as the average increase in CD4 cell count. These measurements inform transition matrices that are used in the model to control patients’ movement between viral load and CD4 cell count health states. Efficacy parameters used in the derivation of transition matrices for each efficacy profile used in the model can be found in Table 5.

Patients can discontinue therapy for virologic (failure, rebound) or non-virologic reasons. Following therapy discontinuation, patients switch to a subsequent therapy determined by the reasons for discontinuation.

Discontinuation due to viral rebound and other non-virologic reasons is managed through treatment-specific transition matrices:

- **Virologic rebound:** Patients who are achieving virologic suppression (viral load <50 copies/mL) face a monthly probability of experiencing virologic rebound. Patients experiencing virologic rebound are assigned to the highest (viral load ≥50 copies/mL) viral load state in a subsequent treatment line.
- **Non-virologic reasons**: Patients in any CD4 or viral load health state face a monthly probability of discontinuing their current line of therapy due to other non-virologic reasons. Patients who discontinue through this process remain in their existing CD4 and viral load health state.

Discontinuation rates can be seen in Table 6.

S1 Table 5. Efficacy parameters for available efficacy profiles

| **Efficacy profile** | **Source** | **Virologic suppression at 48 weeks** | **Baseline CD4 cell count** | **48-week change in CD4 cell count** |
| --- | --- | --- | --- | --- |
|  |  | **Mean (SE)** | **Mean (SD)** | **Mean (SD)** |
| Pooled efficacy | Pooled ATLAS and FLAIR [4] | 93.74% (0.70%) | 671.49 (268.99) | 36.05 (189.37) |
| CAB+RPV LA |  | 93.06% (1.05%) | 672.73 (264.48) | 23.65 (194.64) |
| cART |  | 94.42% (0.94%) | 670.25 (273.42) | 47.82 (183.46) |
| Abbreviations: CAB: cabotegravir; cART: combination antiretroviral therapy; CD4: cluster of differentiation 4; LA: long-acting; RPV: rilpivirine; SD: standard deviation; SE: standard error. | | | | |

S1 Table 6. Probability of virologic, non-virologic and adverse event related discontinuation

| **Efficacy profile** | **Source** | **Time point** | **Virologic discontinuation at 48 weeks** | **Non-virologic discontinuation at 48 weeks** |
| --- | --- | --- | --- | --- |
|  |  |  | **Mean (SE)** | **Mean (SE)** |
| Pooled efficacy | Pooled ATLAS and FLAIR [4] | Year 1 | 0.16% (0.12%) | 0.41% (0.19%) |
|  |  | Year 2 | 0.16% (0.12%) | 0.41% (0.19%) |
|  |  | Year 3+ | 0.16% (0.12%) | 0.41% (0.19%) |
| CAB+RPV LA |  | Year 1 | 0.17% (0.17%) | 0.47% (0.28%) |
|  |  | Year 2 | 0.16% (0.12%) | 0.41% (0.19%) |
|  |  | Year 3+ | 0.16% (0.12%) | 0.41% (0.19%) |
| cART |  | Year 1 | 0.15% (0.16%) | 0.36% (0.25%) |
|  |  | Year 2 | 0.16% (0.12%) | 0.41% (0.19%) |
|  |  | Year 3+ | 0.16% (0.12%) | 0.41% (0.19%) |
| Abbreviations: CAB: cabotegravir; cART: combination antiretroviral therapy; LA: long-acting; RPV: rilpivirine; SE: standard error. | | | | |

- 1. Resistance

Treatment failure while a patient is receiving an ART regimen is frequently associated with the development of resistance. Through appropriate specification of treatment efficacy profiles in each treatment pathway, patients discontinuing treatment due to virologic reasons are assumed to develop resistance which will determine their eligibility for future regimens. For simplicity, additional resistance is not explicitly modelled from third-line treatment onwards.

Patients who have developed the same resistance profile are assumed to receive the same ART in the third line. For example, patients gaining resistance after first-line treatment, but then discontinuing their second-line treatment for non-virologic reasons, and patients who gain resistance after second-line treatment but discontinued their first-line treatment for non-virologic reasons will receive the same third-line ART regimen.

- 1. Safety: adverse events

AEs are incorporated via monthly, treatment-specific probabilities, and are associated with a per event cost. The default inputs relating to the incidence of the most common (incurred by 5% or more of the population) AEs reported in the ATLAS and FLAIR trials can be found in Table 7. AEs are modelled only in the first therapy line.

S1Table 7. First line adverse event incidence

| **AE** | **Pooled (ATLAS and FLAIR)** | | | | **Pooled (ATLAS and FLAIR)** | |
| --- | --- | --- | --- | --- | --- | --- |
|  | **CAB+RPV LA** | | **cART** | | **Pooled efficacy** | |
|  | **Monthly probability (%)** | | | | **Monthly probability (%)** | |
|  | **Mean** | **SE** | **Mean** | **SE** | **Mean** | **SE** |
| Diarrhoea | 0.86% | 0.38% | 0.63% | 0.33% | 0.75% | 0.25% |
| Nasopharyngitis | 1.81% | 0.55% | 1.49% | 0.50% | 1.65% | 0.37% |
| Upper respiratory tract infection | 1.14% | 0.44% | 0.85% | 0.38% | 0.99% | 0.29% |
| Back pain | 0.68% | 0.34% | 0.36% | 0.25% | 0.52% | 0.21% |
| Influenza | 0.67% | 0.33% | 0.54% | 0.30% | 0.60% | 0.22% |
| Cough | 0.41% | 0.26% | 0.41% | 0.26% | 0.41% | 0.19% |
| Pyrexia | 0.68% | 0.34% | 0.20% | 0.18% | 0.44% | 0.19% |
| Fatigue | 0.45% | 0.28% | 0.22% | 0.19% | 0.34% | 0.17% |
| ISR (grade 3/4) | 0.46% | 0.06% | 0.00% | 0.00% | 0.00% | 0.00% |
| Headache | 1.19% | 0.45% | 0.60% | 0.32% | 0.89% | 0.27% |
| ISR (grade 1/2) | 43.31% | 0.41% | 0.00% | 0.00% | 0.00% | 0.00% |
| Respiratory tract infection viral | 0.37% | 0.25% | 0.45% | 0.28% | 0.41% | 0.19% |
| Abbreviations: AE: adverse event; CAB: cabotegravir; cART: combination antiretroviral therapy; ISR: injection site reaction; LA: long-acting; RPV: rilpivirine; SE: standard error  Sources: ATLAS and FLAIR [4]. | | | | | | |

1. Health-related quality of life

Quality of life is incorporated through the application of utility values to each of the CD4 cell count health states included in the Markov component, with utility decrements applied for occurrence of AEs and CVD.

For ADEs, due to a paucity of data relating to OI-specific utility decrements and given that utility decrements associated with ADEs are intrinsically accounted for in the decrements between CD4 cell count states, utility decrements for ADEs were not modelled.

- 1. Health state utility values

Health state utilities are defined by CD4 cell count category for application during all treatment lines. Default model CD4 utility inputs are representative of values published by Kauf et al.[9] (Table 8)[9]; these values were derived from five open-label studies in patients treated with ART.

Utilities are representative of time-point SF-36 measurements, and were estimated as a function of patient demographics, regimen attributes, disease status and AEs using a mixed effects maximum likelihood model.

S1 Table 8. Health state utility values

| **CD4 cell count category (cells/mm^3^)** | **Mean** | **SE** | **Source** |
| --- | --- | --- | --- |
| ≥500 | 0.798 | 0.052 | Kauf (2008)[9] |
| 350–<500 | 0.784 | 0.059 |  |
| 200–<350 | 0.778 | 0.053 |  |
| 50–<200 | 0.750 | 0.058 |  |
| <50 | 0.742 | 0.058 |  |
| Abbreviations: CD4: cluster of differentiation 4; SE: standard error. | | | |

- 1. Age-dependent utility decrement

Age-dependent utility decrements are applied through general population age-dependent utility estimates. Excerpts from age-dependent quality of life data tables are presented in Table 9.

S1 Table 9. Baseline utility specified by age and gender: illustrative values for the UK

| **Age** | **General population utility** | **Source** |
| --- | --- | --- |
| 18 | 0.949 | EuroQol (2014)[10] |
| 19 | 0.946 |  |
| 20 | 0.943 |  |
| . | . |  |
| . | . |  |
| . | . |  |
| 97 | 0.705 |  |
| 98 | 0.702 |  |
| 99 | 0.699 |  |
| 100 | 0.696 |  |
| Note: UK estimates used due to spurious nature of Canadian data; smoothing function fitted to data to extrapolate across all ages. | | |

- 1. Cardiovascular disease utility decrements

Patients developing CVD receive a utility decrement associated with the initial cardiovascular event lasting for one model cycle. Subsequent to this, patients with CVD receive a chronic utility decrement for the remainder of their lives. The values for utility decrements relating to CVD are reported in Table 10.

S1 Table 10. Utility decrements associated with CVD

| **Utility decrement** | **Mean** | **SE** | **Source** |
| --- | --- | --- | --- |
| Initial event | 0.283 | 0.028 | Ara (2009)[11] |
| Chronic | 0.156 | 0.016 |  |
| Abbreviations: SE: standard error  SEs assumed 10% of mean. | | | |

- 1. Adverse event utility decrements

Given the consistent AE profile between therapy arms in the ATLAS and FLAIR trials, with the exception of injection site reactions (ISRs), only a disutility associated with grade 3/4 ISRs has been incorporated (mean: 0.01; SE: 0.001)[4].

1. Mortality

All modelled patients are at risk of all-cause mortality, with health state and the incidence of ADEs and CVD potentially resulting in increased rates of mortality, as described below. Increased rates of mortality are typically applied through the application of relative risks. Relative risks are applied to rates (after conversion from the input probability), before conversion back to probabilities.

- 1. Composite all-cause and adjusted HIV mortality

The model accounts for all-cause mortality using age- and gender-specific mortality rates, derived from country-specific life tables. Once patients reach 100 years in age, they are assumed to die in the next model cycle. The model uses values from 2014-16 Canadian life-tables[12].

S1 Table 11. Excerpt from 2014-16 Canadian life tables

| **Age** | **Male** | **Female** |
| --- | --- | --- |
| 18 | 0.0005 | 0.0003 |
| 19 | 0.0006 | 0.0003 |
| 20 | 0.0006 | 0.0003 |
| … | … | … |
| … | … | … |
| … | … | … |
| 98 | 0.3052 | 0.2596 |
| 99 | 0.3275 | 0.2826 |
| 100 | 0.3498 | 0.3060 |

To reflect the additional mortality in the HIV-1 population, relative risks, stratified by CD4 cell count states (Table 12), are applied to all-cause mortality probabilities.

S1 Table 12. Risk of death relative to all-cause mortality

| **Relative risk of death by CD4 cell count** | **Mean** | **SE** | **Source** |
| --- | --- | --- | --- |
| ≥500 | 2.50 | 1.515 | Lewden (2007)[13] |
| 350-<500 | 3.50 | 1.515 | Lewden (2007)[13] |
| 200-<350 | 5.60 | 0.280 | Lewden (2007)[13] |
| 50-<200 | 30.30* | 0.175* | Lewden (2007)[13] |
| <50 | 30.30* | 0.175* | Lewden (2007)[13] |
| Abbreviations: CD4: cluster of differentiation 4; SE: standard error  * Assumed to be the same as the <200 state in the Lewden study[13]. | | | |

- 1. ADE mortality

Patients experiencing ADEs face an increased risk of mortality. This heightened risk is accounted for using additional ADE-specific mortality probabilities. These monthly probabilities are applied in an additive manner to adjusted all-cause mortality rates and are applied for the duration that the ADE is experienced (ADEs are assumed to last one cycle). Default values, presented in Table 13, were obtained from the ARAMIS DTG Technical report (unpublished data) and are representative of the values derived from the Multicenter AIDS Cohort Study (MACS)[14].

S1 Table 13. AIDS-defining event related mortality

| **Risk of death** | **Mean** | **SE** | **Source** |
| --- | --- | --- | --- |
| Acute viral OI | 0.0492 | 0.0049 | ARAMIS DTG Technical Report (unpublished data);  MACS[14] |
| Acute bacterial OI | 0.0460 | 0.0046 |  |
| Acute fungal OI | 0.0362 | 0.0036 |  |
| Acute protozoan OI | 0.2009 | 0.0201 |  |
| Other OI | 0.0440 | 0.0044 |  |
| Abbreviations: OI: opportunistic infection; SE: standard error. | | | |

1. Costs

Based upon a review of prior HIV cost-effectiveness analyses[1], the model includes costs associated with ART, HIV-specific costs (including OI treatment costs), AE treatment costs, end of life care costs and societal costs.

Costs applied in the model are on either a monthly or per event basis and are discounted at the specified annual discount rates. All costs presented are in 2017 Canadian Dollars.

- 1. Treatment costs

Each treatment is associated with an acquisition cost. Table 14 presents the default costs associated with regimens included in the analysis.

S1 Table 14. ART regimen costs

| **Regimen** | **Monthly cost** | **Source** |
| --- | --- | --- |
| Specified ART regimen | | |
| Pooled comparator | $1,215.76 | Weighted average of ARTs comprising 90% of 2019 Canadian market share |
| CAB+RPV LA | $1,172.85 | Weighted average of integrase inhibitors using 2019 Canadian market share |
| cART | $1,215.76 | Weighted average of ARTs comprising 90% of 2019 Canadian market share |
| Salvage therapy | | |
| Salvage | $2,547.90 | Despiegel et al., (2015) [2] |
| Abbreviations: ART: antiretroviral therapy; CAB: cabotegravir; cART: combination antiretroviral therapy; LA: long acting: RPV: rilpivirine; SE: standard error. | | |

- 1. Additional costs associated with administration of ART

Additional costs are applied to administration of CAB+RPV LA as shown in Table 15 and correspond to an hour of a nurse’s time for the oral initiation cycle, and the first injectable cycle, and fifteen minutes of a nurse’s time for all subsequent injectable cycles.

S1 Table 15. Additional costs associated with administration of CAB+RPV LA

| Cost component | Oral initiation cycle | | First injectable cycle | | Subsequent injectable cycles | | Source |
| --- | --- | --- | --- | --- | --- | --- | --- |
|  | **Mean** | **SE** | **Mean** | **SE** | **Mean** | **SE** |  |
| Health care professional | $35.15 | $3.52 | $35.15 | $3.52 | $8.79 | $0.88 | Ouellet et al (2015)[15] * |
| Abbreviations: SE: standard error  SEs assumed 10% of mean.  *Costs were inflated to 2017 values using The Bank of Canada inflation calculator[16]. | | | | | | | |

- 1. On-treatment adverse event costs

Costs associated with the management of AEs are applied as a per event cost in the cycle of incidence. Given the consistent AE profile between therapy arms in the ATLAS and FLAIR trials, with the exception of ISRs, only a cost associated with ISRs has been incorporated (grade 1/2 [mean: $0.68; SE: $0.068]; grade 3/4 [mean: $191.00; SE: $19.10]) [4].

- 1. HIV-related health encounter costs

Reflective of the additional resource use and healthcare costs associated with HIV-1 infected patients, all-cause health encounter costs have been included across the following resource categories:

- Outpatient visits to HIV primary care provider
- Emergency department visit
- Inpatient days
- CD4 cell count test
- HIV-1 RNA test
- OI prophylaxis
- Resistance testing
- Non-HIV medication

Resource use associated with all-cause health encounters is expected to vary significantly between CD4 cell count health states. As such, the above parameters are stratified by the model’s CD4 cell count health states (CD4 ≥500 cells/mm^3^; CD4 350-<500 cells/mm^3^; CD4 200-<350 cells/mm^3^; CD4 50-<200 cells/mm^3^; CD4 <50 cells/mm^3^).

S1 Table 16. HIV-related health encounter costs

| Variable | CD4 cell count category (cells/mm^3^) | Mean | SE | Source |
| --- | --- | --- | --- | --- |
| Outpatient care | CD4 <50 | $274.63 | $27.46 | Mauskopf (2012)[17] * |
|  | CD4 50-<200 | $234.74 | $23.47 |  |
|  | CD4 200-<350 | $232.84 | $23.28 |  |
|  | CD4 350-<500 | $212.33 | $21.23 |  |
|  | CD4 ≥ 500 | $202.84 | $20.28 |  |
| Non-HIV medication | CD4 <50 | $268.93 | $26.89 |  |
|  | CD4 50-<200 | $165.23 | $16.52 |  |
|  | CD4 200-<350 | $61.91 | $6.19 |  |
|  | CD4 350-<500 | $27.73 | $2.77 |  |
|  | CD4 ≥ 500 | $27.73 | $2.77 |  |
| OI prophylaxis | CD4 <50 | $39.39 | $3.94 | Ontario Drug Benefit Formulary[18] |
|  | CD4 50-<200 | $13.78 | $1.38 |  |
|  | CD4 200-<350 | $0.00 | $0.00 | NA |
|  | CD4 350-<500 | $0.00 | $0.00 | NA |
|  | CD4 ≥ 500 | $0.00 | $0.00 | NA |
| Abbreviations: CD4: cluster of differentiation 4; HIV-1: human immunodeficiency virus type 1; NA: not applicable; OI: opportunistic infection; RNA: ribonucleic acid; SE: standard error  SEs assumed 10% of mean  * Costs were inflated to 2017 values using The Bank of Canada inflation calculator[16]. | | | | |

- 1. Cardiovascular disease-related costs

The incidence of CVD is assumed to incur both an initial one-off event cost, applied when CVD is first experienced, and a subsequent monthly cost applied on a cyclical basis. The inputs, presented in Table 17, were inflated and transformed to monthly costs.

- 1. Cost of death

End of life care costs are reflective of the additional resource use incurred by individuals in the months prior to death and are applied in the final month of life, these are presented in Table 17.

- 1. AIDS-defining event costs

The costs associated with the treatment of OIs are applied in the model as per event costs in the cycle of incidence and have been presented in Table 17. These values were derived from a study by Anis et al.[19], which examined the cost-effectiveness of different ART regimens in a Canadian setting[19].

S1 Table 17. Costs associated with CVD, death and ADEs

| Cost variable | Mean | SE | Source |
| --- | --- | --- | --- |
| CVD costs | | | |
| Initial cost associated with event | $6,365.79 | $636.58 | Akerborg (2012)[20] |
| Additional monthly costs applied in each subsequent month | $1,602.77 | $160.28 | Akerborg (2012)[20] |
| Cost of death | | | |
| End of life care cost (last 3-months) | $20,976.97 | $2,097.70 | Canadian Institute for Health Information (2008)[21] |
| Cost of ADE | | | |
| Acute viral OI | $5,793.36 | $579.34 | Anis (2000)[19] |
| Acute bacterial OI | $5,130.73 | $513.07 |  |
| Acute fungal OI | $5,660.80 | $566.08 |  |
| Acute protozoan OI | $5,454.92 | $545.49 |  |
| Other OI | $6,652.57 | $665.26 |  |
| Abbreviations: ADE: AIDS-defining event; CVD: cardiovascular disease; OI: opportunistic infection; SE: standard error  SEs assumed 10% of mean  * Cost inputs were inflated to 2017 values using The Bank of Canada inflation calculator[16]. | | | |

1. Sensitivity analysis

In the sensitivity analysis key parameters from both the cost-effectiveness model and viral transmission model were varied, with the impact of the most influential parameters on key model outputs reported. The full list of parameters varied is presented below.

Parameters from the cost-effectiveness model

- Treatment duration without response (3 months)
- Risk of death relative to all-cause mortality (+/- 20%)
- Probability of CAB+RPV LA virologic discontinuation (+/- 20%)
- Probability of oral cART virologic discontinuation (+/- 20%)
- Percentage of cohort that are female (0-100%)
- Health state utilities (+/- 20%)
- CAB+RPV LA acquisition cost (+/- 10%)
- Costs discount rate (0% - 3%)
- Benefits discount rate (0% - 3%)
- Average baseline age of cohort (+/- 20%)
- ADE costs (+/- 20%)
- All-cause health encounter costs (+/- 20%)

Parameters from the viral transmission model

- Proportion of population in each of the following populations:
  - Low risk heterosexual (+/- 20%)
  - High risk heterosexual (+/- 20%)
  - MSM transmission risk (+/- 20%)
  - MSM & IDU transmission risk (+/- 20%)
  - Heterosexual & IDU transmission risk (+/- 20%)
- Each of the following parameters in each of the low-risk heterosexual, high-risk heterosexual and MSM populations:
  - Average partnership duration (+/- 20%)
  - Number of sexual acts per partner per month (+/- 20%)
  - Probability of condom use (+/- 20%)
  - Transmission hazard ratio for condom use (+/- 20%)
  - Probability of transmission per sexual act (+/- 20%)
- Injection drug use parameters:
  - Monthly injection frequency (+/- 20%)
  - Probability of shared injection (+/- 20%)
  - Probability of opioid agonist treatment (+/- 20%)
  - Transmission hazard ratio for opioid agonist treatment (+/- 20%)
  - Probability of transmission per shared injection (+/- 20%)

1. Validation

Demonstrating the validity and credibility of health economic models is a vital component in ensuring their adequacy to support health economic decision making[22]. Previous iterations of the model have undergone external consistency validation exercises, designed to demonstrate that values predicted by the cost-effectiveness model are consistent with previously published outcomes. Details of the external validation are provided below, with validation exercises also presented at ISPOR conference proceedings[23-25].

- 1. External validity
     1. Method

Studies were identified from a review of previous cost-effectiveness studies in HIV[1], in addition to a search of grey literature. Studies that used models whose structure differed significantly from the CAB+RPV LA model were excluded; i.e., only models with structures that the CAB+RPV LA model could adequately replicate (minimum four therapy lines, three definable ART lines and a salvage therapy line) were considered in the validation. Studies providing insufficient information regarding model inputs were also excluded.

For each validation exercise, model inputs (demographics, baseline risk factors, HIV disease status, costs and quality of life values) corresponding to published profiles were entered into the CAB+RPV LA cost-effectiveness model workbook. Where required model inputs were not reported, default model inputs were used, or reasonable assumptions were made.

Details of included studies and assumptions made can be found in Table 18.

S1 Table 18. Validation studies and associated assumptions

| Study | Assumptions/Notes |
| --- | --- |
| Despiegel (2015)[2]  Cost-Effectiveness of Dolutegravir in HIV-1 Treatment Naïve and Treatment-Experienced Patients in Canada. | - Baseline viral status: Initial health state distribution was imputed by fitting normal distributions to mean and SD values to the CD4 and viral load values presented for the initiated cohort. - Framingham risk equation: Lipid parameters assumed constant to those presented at baseline. - Mortality relative risks: Relative risk factors associated with CVD were assumed as model defaults. - CVD: initial cost and utility decrement assumed as model defaults. |
| Brogan (2014)[26]  Cost Effectiveness of Darunavir/ritonavir Combination Antiretroviral Therapy for Treatment-Naïve adults with HIV-1 Infection in Canada. | - Baseline viral status: The initial health state distribution of the cohort was assumed equal to the values derived by imputing a normal distribution using mean and SD viral load values presented in the ARTEMIS trial and by multiplying by the proportion of the cohort in each CD4 health state at baseline. |
| Brogan (2011)[27] Cost-Effectiveness of Nucleoside Reverse Transcriptase Inhibitor Pairs in Efavirenz-Based Regimens for Treatment-Naïve Adults with HIV Infection in the United States | - Baseline viral status: Initial health state distribution was imputed by fitting normal distributions to mean and SD values to the CD4 and viral load values presented for the initiated cohort. - Transition matrices: Derived based on patient virologic response at 48 weeks. - Mortality: Relative risk applied to all-cause mortality equally across all CD4 cell count states. Additional mortality as a result of HIV infection modelled through AIDS-defining events. |
| Walenksy (2013)[28]  Economic Savings Versus Health Losses: The Cost-Effectiveness of Generic Antiretroviral Therapy in the United States | - Mortality relative risks: Relative risk factors associated with CD4 cell count were assumed as model defaults. - AIDS-defining events: Probability of experiencing AIDS-defining events and associated mortality assumed as model defaults. - CD4 state utility: Utility values associated with CD4 cell count states were assumed as model defaults. |
| Peng (2015)[29]  Cost-effectiveness of DTG + ABC/3TC versus EFV/ TDF/FTC for first-line treatment of HIV-1 in the United States | - CD4 state utility: Utility values associated with CD4 cell count states were assumed as model defaults. |
| Abbreviations: CD4: cluster of differentiation 4; CVD: cardiovascular disease; SD: standard deviation. | |

- 1. Goodness of fit

For each validation exercise, predicted values for total costs, QALYs and ICERs, were compared with the published (expected) values. Consistent with previously published health economic validation studies, goodness of fit was measured using the coefficient of determination (R^2^), mean absolute percentage error (MAPE) and the root mean square percentage error (RMSPE). MAPE and RMSPE were calculated using the following equations:

$$MAPE= \frac{1}{n}\sum_{\dot{I}=1}^{n} \left| \left( \frac{Y_{i}-X_{i}}{Y_{i}} \right)\times100 \right|$$

$$RMSPE \sqrt{\frac{\sum_{i=1}^{n} \left( X_{i}-Y_{i} \right)^{2}}{n}}$$

In which, X_1_, X_2_,…, X_n_ correspond to endpoints as predicted by the HIV CAB+RPV LA cost-effectiveness model, Y_1_, Y_2_,… Y_n_  correspond to endpoints as observed in the published literature, where *n* represents the sample size (i.e. the number of validation endpoints). The residuals *Z* are defined as the differences between the two outcomes: For i = 1,2,…,n, Z = Y – X for.

Further, we present a scatterplot of the observed versus predicted outcomes along the coefficient of determination.

- 1. Results

With an Overall R^2^ value of 0.937, a high degree of linear correlation is observed between predicted and expected endpoints. Further, with RMSPE and MAPE values of 14.7% and 17.7%, respectively, the HIV CAB+RPV LA cost-effectiveness model exhibits a high degree of consistency with previously published cost-effectiveness analyses.

A graphical representation of the relationship between the observed and predicted endpoints of the individual studies has been presented in Fig 4.


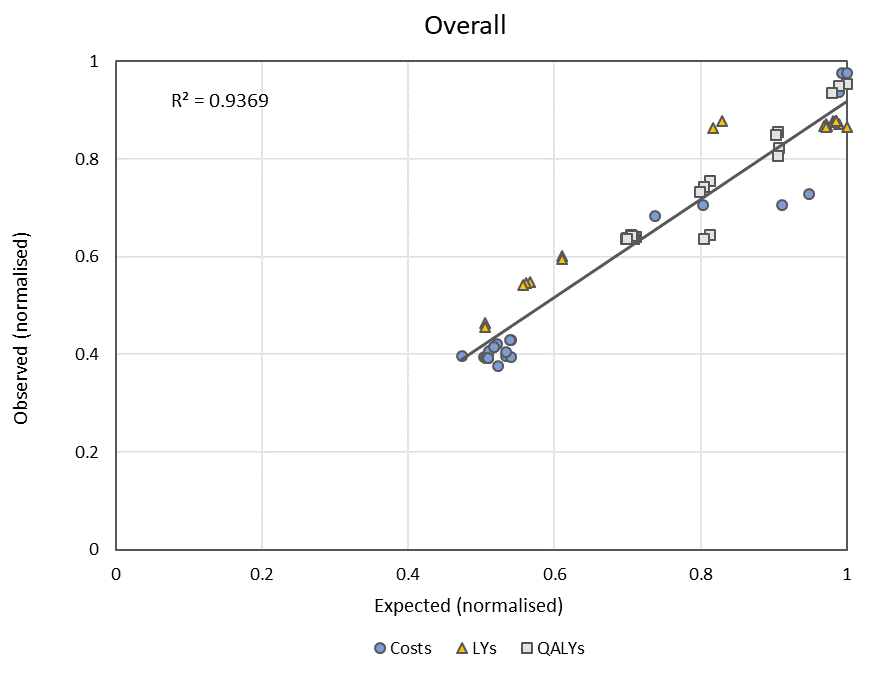


S1 Fig 4. Normalised observed (model predicted) versus expected (published results) validation results for costs, life years (LYs) and quality adjusted life years (QALYs) from the HIV CAB+RPV LA cost-effectiveness model

# **Appendix A.**

**Framingham risk equation**

Cardiovascular event risk is modelled using a sex-specific Cox proportional hazards model developed from the Framingham study cohort.[8] The risk of developing CVD is considered to be a function of age, total cholesterol, High-density lipoprotein cholesterol, systolic blood pressure, anti-hypertensive medication use, smoking and diabetic status.

The general formula utilised in the derivation of CVD risk is:

$\hat{p}$ *= 1-* $S_{0}{(t)}^{exp(\Sigma_{ⅈ=1}^{p}\beta_{i}X_{i} - \Sigma_{i=1}^{p}\beta_{i} \bar{X}_{i})}$

In which, S_0_(t) is the baseline survival at follow-up time *t,* β_i_ is the estimated regression coefficient (the natural log of the hazard ratio), *X_i_* is the log-transformed value of the *i^th^* variable, $\bar{X}_{i}$ is the corresponding mean, and *p* denotes the number of risk factors.

A summary of the coefficients used by the model to derive 10-year CVD risk is presented in Table 19.

S1 Table 19. Framingham regression coefficients and hazard ratios

| Variable | β*** | P-value | Hazard ratio |
| --- | --- | --- | --- |
| Women [S_0_(10) = 0.95012] | | | |
| Log of age | 2.32888 | <0.001 | 10.27 |
| Log of total cholesterol | 1.20904 | <0.001 | 3.35 |
| Log of HDL cholesterol | -0.70833 | <0.001 | 0.49 |
| Log of SBP if not treated | 2.76157 | <0.001 | 15.82 |
| Log of SBP if treated | 2.82263 | <0.001 | 16.82 |
| Smoking | 0.52873 | <0.001 | 1.70 |
| Diabetes | 0.69154 | <0.001 | 2.00 |
| Men [S_0_(10) = 0.88936] | | | |
| Log of age | 3.06117 | <0.001 | 21.35 |
| Log of Total cholesterol | 1.12370 | <0.001 | 3.08 |
| Log of HDL cholesterol | -0.93263 | <0.001 | 0.39 |
| Log of SBP if not treated | 1.93303 | <0.001 | 6.91 |
| Log of SBP if treated | 1.99881 | <0.001 | 7.38 |
| Smoking | 0.65451 | <0.001 | 1.92 |
| Diabetes | 0.57367 | <0.001 | 1.78 |
| Abbreviations: SBP: systolic blood pressure; HDL: high-density lipoproteins  S_0_ indicates 10-year baseline survival  *Estimated regression coefficient. | | | |

References

1. Mauskopf J. A methodological review of models used to estimate the cost effectiveness of antiretroviral regimens for the treatment of HIV infection. PharmacoEconomics. 2013;31(11):1031-50.

2. Despiégel N, Anger D, Martin M, Monga N, Cui Q, Rocchi A, et al. Cost-Effectiveness of Dolutegravir in HIV-1 Treatment-Naive and Treatment-Experienced Patients in Canada. Infectious diseases and therapy. 2015;4(3):337-53.

3. Baril JG, Angel JB, Gill MJ, Gathe J, Cahn P, van Wyk J, et al. Dual Therapy Treatment Strategies for the Management of Patients Infected with HIV: A Systematic Review of Current Evidence in ARV-Naive or ARV-Experienced, Virologically Suppressed Patients. PloS one. 2016;11(2):e0148231.

4. Rizzardini Giuliano, Overton Edgar T, Orkin Chloe, et al. Long-Acting Injectable Cabotegravir+ Rilpivirine for HIV Maintenance Therapy: Week 48 Pooled Analysis of Phase 3 ATLAS and FLAIR Trials. JAIDS Journal of Acquired Immune Deficiency Syndromes

5. Kanters S, Socias ME, Paton NI, Vitoria M, Doherty M, Ayers D, et al. Comparative efficacy and safety of second-line antiretroviral therapy for treatment of HIV/AIDS: a systematic review and network meta-analysis. The Lancet HIV. 2017.

6. Samji H, Taha TE, Moore D, Burchell AN, Cescon A, Cooper C, et al. Predictors of unstructured antiretroviral treatment interruption and resumption among HIV-positive individuals in Canada. HIV medicine. 2015;16(2):76-87.

7. Ross EL, Weinstein MC, Schackman BR, Sax PE, Paltiel AD, Walensky RP, et al. The clinical role and cost-effectiveness of long-acting antiretroviral therapy. Clinical infectious diseases : an official publication of the Infectious Diseases Society of America. 2015;60(7):1102-10.

8. D’Agostino RB, Vasan RS, Pencina MJ, Wolf PA, Cobain M, Massaro JM, et al. General cardiovascular risk profile for use in primary care. Circulation. 2008;117(6):743-53.

9. Kauf TL, Roskell N, Shearer A, Gazzard B, Mauskopf J, Davis EA, et al. A predictive model of health state utilities for HIV patients in the modern era of highly active antiretroviral therapy. Value in Health. 2008;11(7):1144-53.

10. Self-Reported Population Health: An International Perspective based on EQ-5D. In: Szende A, Janssen B, Cabases J, editors. Dordrecht: Springer; 2014.

11. Ara R, Brazier J. Health related quality of life by age, gender and history of cardiovascular disease: results from the Health Survey for England2009 16 August 2017. Available from: <https://www.sheffield.ac.uk/scharr/sections/heds/discussion-papers/912-1.292427>.

12. Statistics Canada. Life expectancy and other elements of the life table, Canada, all provinces excpet Prince Edward Island,2014-2016 23.11.2018. Available from: <https://www150.statcan.gc.ca/t1/tbl1/en/tv.action?pid=1310011401&pickMembers%5B0%5D=1.1&pickMembers%5B1%5D=3.2&pickMembers%5B2%5D=4.3>,.

13. Lewden C, Chêne G, Morlat P, Raffi F, Dupon M, Dellamonica P, et al. HIV-infected adults with a CD4 cell count greater than 500 cells/mm3 on long-term combination antiretroviral therapy reach same mortality rates as the general population. JAIDS Journal of Acquired Immune Deficiency Syndromes. 2007;46(1):72-7.

14. Rydzak CE, Cotich KL, Sax PE, Hsu HE, Wang B, Losina E, et al. Assessing the performance of a computer-based policy model of HIV and AIDS. PloS one. 2010;5(9):e12647.

15. Ouellet E, Durand M, Guertin JR, LeLorier J, Tremblay CL. Cost effectiveness of ‘on demand’HIV pre-exposure prophylaxis for non-injection drug-using men who have sex with men in Canada. Canadian Journal of Infectious Diseases and Medical Microbiology. 2015;26(1):23-9.

16. The Bank of Canada: Banque du Canada. Inflation Calculator16 August 2017. Available from: <http://www.bankofcanada.ca/rates/related/inflation-calculator/>.

17. Mauskopf J, Brogan AJ, Talbird SE, Martin S. Cost-effectiveness of combination therapy with etravirine in treatment-experienced adults with HIV-1 infection. AIDS (London, England). 2012;26(3):355-64.

18. Ontario Drug Benefit Formulary/Comparative Drug Index [Internet]. [cited 16 August 2017]. Available from: <https://www.formulary.health.gov.on.ca/>.

19. Anis AH, Guh D, Hogg RS, Wang X-H, Yip B, Craib KJ, et al. The cost effectiveness of antiretroviral regimens for the treatment of HIV/AIDS. PharmacoEconomics. 2000;18(4):393-404.

20. Akerborg O, Nilsson J, Bascle S, Lindgren P, Reynolds M. Cost-effectiveness of dronedarone in atrial fibrillation: results for Canada, Italy, Sweden, and Switzerland. Clinical therapeutics. 2012;34(8):1788-802.

21. Canadian Institute for Health Information. Health Indicators2008 [22nd November 2018]. Available from: <https://secure.cihi.ca/free_products/HealthIndicators2008_ENGweb.pdf>.

22. Eddy DM, Hollingworth W, Caro JJ, Tsevat J, McDonald KM, Wong JB. Model transparency and validation: a report of the ISPOR-SMDM Modeling Good Research Practices Task Force–7. Medical Decision Making. 2012;32(5):733-43.

23. Darlington O, Lopes, S., Ward, T., Surgue, D., McEwan, P., Martin, A.A., Punekar, Y.S.,. PIN55 Estimating health outcomes in HIV infected patients treated with a dual regimen of dolutegravir and rilpivirine in Europe, . Presented at ISPOR US 2018,; Baltimore, US2018.

24. McEwan P, Darlington, O., van Doornewaard, A., Webster, S., Ward, T., Martin, A., Punekar, Y.,. PIN63, Estimating Life Years and Quality-Adjusted Life Years in Heavily Treatment-Experienced (HTE) Patients,. Presented at ISPOR Europe 2017,; Glasgow, Scotland, 4-8 November 20172017.

25. Ward T, Punekar, Y., Darlington, O., McEwan, P., Martin, A., Urbaityte, R., Lopes, S.,. PIN64, Dolutegravir plus lamivudine for the treatment of naïve adults living with HIV-1: A UK cost-minimization analysis. Presented at ISPOR Europe 2018,; Barcelona, Spain, 10-14 November 20182018.

26. Brogan AJ, Smets E, Mauskopf JA, Manuel SA, Adriaenssen I. Cost effectiveness of darunavir/ritonavir combination antiretroviral therapy for treatment-naive adults with HIV-1 infection in Canada. PharmacoEconomics. 2014;32(9):903-17.

27. Brogan AJ, Talbird SE, Cohen C. Cost-effectiveness of nucleoside reverse transcriptase inhibitor pairs in efavirenz-based regimens for treatment-naive adults with HIV infection in the United States. Value in Health. 2011;14(5):657-64.

28. Walensky RP, Sax PE, Nakamura YM, Weinstein MC, Pei PP, Freedberg KA, et al. Economic savings versus health losses: the cost-effectiveness of generic antiretroviral therapy in the United States. Annals of internal medicine. 2013;158(2):84-92.

29. Peng S, Tafazzoli A, Dorman E, Rosenblatt L, Villasis-Keever A, Sorensen S. Cost-effectiveness of DTG+ ABC/3TC versus EFV/TDF/FTC for first-line treatment of HIV-1 in the United States. Journal of medical economics. 2015;18(10):763-76.
