# Supplementary material for: Cost-effectiveness of the long-acting regimen cabotegravir plus rilpivirine for the treatment of HIV-1 and its potential impact on adherence and viral transmission: A modelling study
Source: PLoS One. 2021 Feb 2;16(2):e0245955. doi: 10.1371/journal.pone.0245955 (PMC7853524; doi:10.1371/journal.pone.0245955)
Supplement: S2 Appendix — (DOCX) [file pone.0245955.s002.docx]

**S2 Appendix: viral transmission**

1. **Model design**
   1. Structure
      1. Overview

The viral transmission module utilises data estimated through the cost-effectiveness model, alongside viral transmission parameters to estimate the total number of onwards infections attributable to the modelled cohort. Outcomes from both the modelled cohort and the onwards transmissions are subsequently evaluated in terms of cost-effectiveness. An overview of the model process is provided in Figure 1.


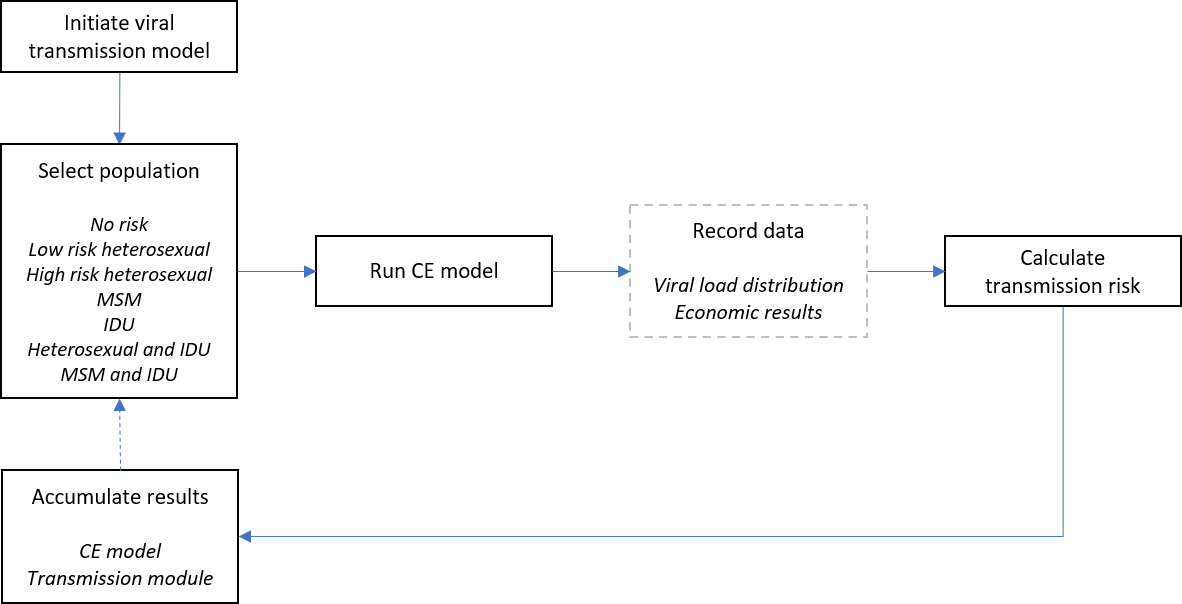


**Supplementary Figure 1.** Overview of model process

Patients are stratified into one of several risk groups, with the majority of risk groups potentially contributing to onwards transmission of HIV. Each population is described in detail in Table 1. Based on these core transmission risk populations, a conceptual model design was developed (Figure 2). The heterosexual risk population was further stratified into low-risk and high-risk behaviour categories and transmission from multiple sources was permitted (e.g. heterosexual transmission in the injecting drug user (IDU) transmission risk group).

**Supplementary Table 1. Population stratification**

| Population | | Description | Transmission |
| --- | --- | --- | --- |
| No risk of onwards transmission | | Patients are assumed to never participate in behaviour that may lead to onwards HIV transmission | Patients may not infect others during the modelled time horizon |
| Heterosexual transmission risk* | Low risk | Patients that are at risk of transmitting HIV through sexual intercourse with members of the opposite sex; stratified into high risk (e.g. sex workers) and low risk (e.g. general population) groups | Patients incur a time-dependent risk of infecting their sexual partners during periods of high viral load |
|  | High risk |  |  |
| MSM transmission risk* | | Male patients that are at risk of transmitting HIV through sexual intercourse with members of the same sex | Patients incur a time-dependent risk of infecting their sexual partners during periods of high viral load |
| IDU transmission risk* | | Patients that are at risk of transmitting HIV through injection drug use and needle sharing | Patients incur a time-dependent risk of infecting those that they share injecting equipment with during periods of high viral load |
| Abbreviations: IDU: injecting drug users; MSM: men who have sex with men  *Cross-population transmission is possible (i.e. IDU patients transmitting HIV through sexual intercourse). | | | |

The modelled cohort is initially distributed across each of the risk groups. The cost-effectiveness model is subsequently used to estimate lifetime outcomes for each of the risk groups. Lifetime costs, life years (LYs) and quality adjusted life years (QALYs) associated with HIV viral progression in the modelled cohort are accrued and recorded.

To estimate the number of onwards infections, the lifetime viral load health state occupancy of each risk group is recorded. It is assumed that the modelled cohort may only contribute to onwards HIV infections if they have a viral load ≥50 copies/mL, therefore it is only during periods of high viral load that patients may infect others. Subsequently, time spent in the higher viral load states is combined with the time-dependent risk of transmission (based on risk group-specific behaviour characteristics) to estimate the number of onwards HIV infections attributed to the initial cohort.

Total lifetime costs, LYs and QALYs for each onwards infection are estimated and incorporated within the initial cohort cost-effectiveness calculation. Only direct infections are considered within the model.
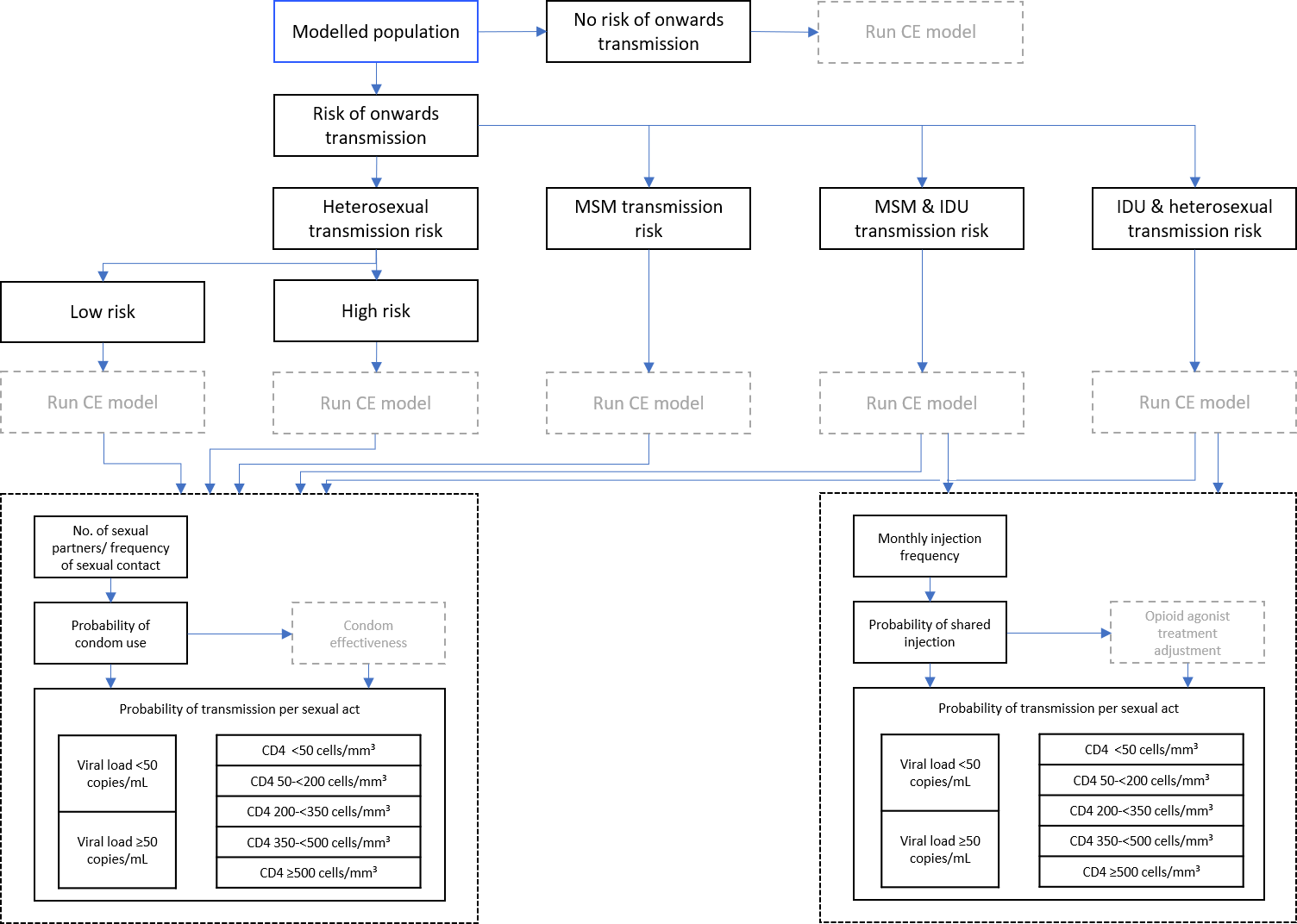


**Supplementary Figure 2.** Viral transmission module flow diagram

- - 1. Patient characteristics

Upon initiation of the viral transmission module, the cost-effectiveness model is run for each of the transmission risk groups and outcomes relevant to the calculation of onwards transmissions are recorded.

- - 1. **Transmission risk**

Transmission risk is defined individually within each risk group and is derived from a series of behaviour-related parameters.

- - - 1. Sexual transmission

The transmission risk associated with sexual transmission is derived from the following key pieces of information:

- Expected number/duration of sexual partnerships
- Mean number of sex acts per-partner per-month
- Likelihood of condom use
- The risk of transmission per sex act, stratified by viral load and cluster of differentiation 4 (CD4) health state

The expected number/duration of sexual partnerships is primarily utilised to limit the number of transmissions attributed to each patient, with the assumption that patients may not have more than one partner at a time, and that they may only infect their current partner.

Sexual partnerships are modelled continuously over the patient’s lifetime and during each sexual partnership the modelled patient may infect only their current partner. The model utilises the above parameters to calculate the risk of transmission over a given partnership. A reduction in the likelihood of risky sexual behaviour over a patient’s lifetime is captured through the incorporation of age-dependent hazard ratios (HRs) applied to the number of monthly sex acts. Age-dependent HRs are available for the following age groups:

- ≤30 years
- 30-40 years
- 40-50 years
- 50-60 years
- >60 years

Additional adjustment to the likelihood of sexual transmission may be made through estimation of the proportion of sexual acts in which a condom was used. Such acts then observe a reduction in the risk of onwards transmission.

The risk of transmission per sexual act is calculated using the following equation:

*P(Transmission by VL/CD4) x (P(condom use) x Condom use HR + (1 – P(condom use))*

Subsequently, the likelihood of a patient infecting their current partner in any given month is calculated using the following equation:

*(1 – ((1 – P(transmission per sex act)) ^ (Monthly no. sex acts x Age-related behaviour HR))*

- - - 1. IDU transmission

The transmission risk associated with IDU transmission is derived from the following parameters:

- Mean monthly injection frequency
- Likelihood of an injection being shared
- Likelihood of opioid agonist treatment (OAT)
- The risk of transmission per shared injection, stratified by viral load and CD4 health state

IDU is modelled continuously over the patient’s lifetime and during each month, the number of onwards transmissions may be limited. The model utilises the above parameters to calculate the risk of transmission over a patient’s lifetime. A reduction in the number of shared injections over a patient’s lifetime is captured through the incorporation of age-dependent HRs applied to the number of monthly shared injections.

Additional adjustment to the likelihood of IDU transmission may be made through estimation of the proportion of patients initiating OAT. Such therapy initiation results in a reduction in the risk of onwards transmission.

The risk of transmission per shared injection is specified for each viral load health state and, subsequently, the number of infections incurred through IDU is calculated using the following equation:

*(1 – ((1 – P(transmission per shared injection)) ^ (Monthly no. injections x P(shared injection) x Age-related behaviour HR x (P(OAT) x OAT HR + (1 – P(OAT))))*

1. Key model assumptions

A list of key model assumptions is provided in Table 2.

**Supplementary Table 2. Key model assumptions**

| Assumption | Notes |
| --- | --- |
| Under default settings, patients with a viral load of <50 copies/mL do not contribute to onwards transmission, however the functionality to model this is incorporated if required | Supported by published data[1] |
| All partners/contacts are unique/independent | A simplifying assumption that means patients can’t infect the same person; potential to overestimate onwards transmission |
| Transmission from multiple sources is applied additively (e.g. IDU transmission is applied to a different pool of susceptible patients than sexual transmission) | An assumption required due to a lack of published data |
| Sexual transmission risk in IDU patients utilises parameters from the low risk heterosexual population | Assumed due to greater prevalence of low risk heterosexual population |
| Sexual partnerships are modelled continuously (i.e. time without a partner is not modelled) | A simplifying assumption, however, default parameters are based on number of sexual partners in the past 12 months and so this methodology remains appropriate |
| During any given partnership, patients only interact with one sexual partner | Assumption applied due to a lack of published data |
| Vertical transmission is not included in the analysis | Mother to child transmission is relatively rare in Canada and unlikely to impact results significantly |
| Patients in the IDU transmission risk group may infect more than one person per-month | It is assumed that IDU patients may share injecting equipment with multiple people |
| Each sexual act and injection incur the same likelihood of transmission | Assumption in line with previous modelling studies |
| Abbreviations: IDU: injecting drug users | |

1. Model inputs

Model input parameters are described in Table 3 to Table 7.

**Supplementary Table 3. Model input parameters: cohort distribution**

| Parameter | Value | Source |
| --- | --- | --- |
| No risk of transmission | 0.00% | Public Health Agency of Canada[2] |
| Non-IDU transmission | 81.51% |  |
| Heterosexual transmission | 32.20% |  |
| Low risk | 28.98% |  |
| High risk | 3.22% |  |
| MSM transmission | 49.31% |  |
| IDU transmission | 18.49% |  |
| MSM & IDU | 3.18% |  |
| Heterosexual & IDU | 15.31% |  |
| Abbreviations: IDU: injecting drug users; MSM: men who have sex with men. | | |

**Supplementary Table 4. Model input parameters: sexual transmission risk behaviour**

| Parameter | Heterosexual | | | | MSM | |
| --- | --- | --- | --- | --- | --- | --- |
|  | **Low risk** | | **High risk** | |  |  |
|  | **Value** | **Source** | **Value** | **Source** | **Value** | **Source** |
| Average partnership duration | 11.99 | Haderxhanaj et al.[3] | 0.06 | Brewer et al.[4] | 4.00 | Glick et al.[5] |
| Age at which no further new partners observed | 70.00 | Assumed | 70.00 | Assumed | 70.00 | Assumed |
| Probability of condom use | 26.00% | Nosyk et al.[6] | 62.76% | Campeau et al.[7] | 46.00% | Nosyk et al.[6] |
| Transmission HR for condom use | 0.1 | Nosyk et al.[6] | 0.1 | Nosyk et al.[6] | 0.1 | Nosyk et al.[6] |
| Monthly no. sexual acts | 4.48 | Twenge et al.[8] | 1.00 | Assumed | 8.33 | Remis et al.[9] |
| Age behaviour risk HR | | | | | | |
| ≤30 years | 1.000 | Twenge et al.[8] | 1.000 | Assumed as low risk heterosexual | 1.000 | Assumed as low risk heterosexual |
| 30-40 years | 0.919 |  | 0.919 |  | 0.919 |  |
| 40-50 years | 0.721 |  | 0.721 |  | 0.721 |  |
| 50-60 years | 0.471 |  | 0.471 |  | 0.471 |  |
| >60 years | 0.234 |  | 0.234 |  | 0.234 |  |
| Abbreviations: HR: hazard ratio; MSM: men who have sex with men. | | | | | | |

**Supplementary Table 5. Model input parameters: IDU transmission risk behaviour**

| Parameter | Value | Source |
| --- | --- | --- |
| Monthly injection frequency | 19.00 | Nosyk et al.[6] |
| Probability of shared injection | 1.30% | Nosyk et al.[6] |
| Probability of OAT | 31.30% | Nosyk et al.[6] |
| Transmission HR for OAT | 0.75 | Nosyk et al.[6] |
| Age behaviour risk HR | | |
| ≤30 years | 1.000 | Assumed |
| 30-40 years | 1.000 |  |
| 40-50 years | 1.000 |  |
| 50-60 years | 1.000 |  |
| >60 years | 0.000 |  |
| Abbreviations: HR: hazard ratio; IDU: injection drug use; OAT: opioid agonist treatment. | | |

**Supplementary Table 6. Model input parameters: probabilities of transmission**

| Parameter | Heterosexual | | MSM | | IDU | |
| --- | --- | --- | --- | --- | --- | --- |
|  | **Value** | **Source** | **Value** | **Source** | **Value** | **Source** |
| Viral load ≥50 copies/mL | | | | | | |
| CD4<50 | 0.044% | Public Health Agency of Canada[2]  Hughes et al.[10]  Wilson et al.[11] | 0.470% | Public Health Agency of Canada[2]  Baggaley et al.[12]  Boily et al.[13]  Fox et al.[14]  Jin et al.[15]  Vitinghoff et al.[16] | 0.300% | Public Health Agency of Canada[2]  Lee et al.[17] |
| CD4 50-<200 | 0.044% |  | 0.470% |  | 0.300% |  |
| CD4 200-<350 | 0.044% |  | 0.470% |  | 0.300% |  |
| CD4 350-<500 | 0.044% |  | 0.470% |  | 0.300% |  |
| CD4 ≥500 | 0.044% |  | 0.470% |  | 0.300% |  |
| Abbreviations: CD4: cluster of differentiation 4; HR: hazard ratio; MSM: men who have sex with men. | | | | | | |

**Supplementary Table 7. Model input parameters: lifetime economic inputs**

| Parameter | Value | Source |
| --- | --- | --- |
| Discounted outcomes of newly infected HIV patient | | |
| Total lifetime HIV cost | 997,422 | Brogan et al.[18] |
| Total LYs (from diagnosis) | 26.28 | Assumed based on Brogan et al.[18] |
| Total QALYs (from diagnosis) | 21.64 | Brogan et al.[18] |
| Discounted outcomes of non-HIV infected person | | |
| Total lifetime HIV cost | 0.00 | Assumed |
| Total LYs (from diagnosis) | 33.72 | Statistics Canada[19] |
| Total QALYs (from diagnosis) | 27.77 | Szende et al.[20] |
| Abbreviations: LY: life year; QALY: quality-adjusted life year. | | |

References

1. Rodger AJ, Cambiano V, Bruun T, Vernazza P, Collins S, Van Lunzen J, et al. Sexual activity without condoms and risk of HIV transmission in serodifferent couples when the HIV-positive partner is using suppressive antiretroviral therapy. Jama. 2016;316(2):171-81.

2. Public Health Agency of Canada. Summary: Estimates of HIV incidence, prevalence and proportion undiagnosed in Canada, 201401 June 2018. Available from: <https://www.canada.ca/en/public-health/services/publications/diseases-conditions/summary-estimates-hiv-incidence-prevalence-proportion-undiagnosed-canada-2014.html>.

3. Haderxhanaj LT, Leichliter JS, Aral SO, Chesson HW. Sex in a lifetime: Sexual behaviors in the United States by lifetime number of sex partners, 2006–2010. Sexually transmitted diseases. 2014;41(6):345-52.

4. Brewer DD, Potterat JJ, Garrett SB, Muth SQ, Roberts JM, Kasprzyk D, et al. Prostitution and the sex discrepancy in reported number of sexual partners. Proceedings of the National Academy of Sciences. 2000;97(22):12385-8.

5. Glick SN, Morris M, Foxman B, Aral SO, Manhart LE, Holmes KK, et al. A comparison of sexual behavior patterns among men who have sex with men and heterosexual men and women. Journal of acquired immune deficiency syndromes (1999). 2012;60(1):83.

6. Nosyk B, Min JE, Lima VD, Hogg RS, Montaner JS. Cost-effectiveness of population-level expansion of highly active antiretroviral treatment for HIV in British Columbia, Canada: a modelling study. The lancet HIV. 2015;2(9):e393-400.

7. Campeau L, Blouin K, Leclerc P, Alary M, Morissette C, Blanchette C, et al. Impact of sex work on risk behaviours and their association with HIV positivity among people who inject drugs in Eastern Central Canada: cross-sectional results from an open cohort study. BMJ open. 2018;8(1):e019388.

8. Twenge JM, Sherman RA, Wells BE. Declines in sexual frequency among American adults, 1989–2014. Archives of sexual behavior. 2017;46(8):2389-401.

9. Remis RS, Alary M, Liu J, Kaul R, Palmer RW. HIV transmission among men who have sex with men due to condom failure. PloS one. 2014;9(9):e107540.

10. Hughes JP, Baeten JM, Lingappa JR, Magaret AS, Wald A, De Bruyn G, et al. Determinants of per-coital-act HIV-1 infectivity among African HIV-1–serodiscordant couples. Journal of Infectious Diseases. 2012;205(3):358-65.

11. Wilson DP, Law MG, Grulich AE, Cooper DA, Kaldor JM. Relation between HIV viral load and infectiousness: a model-based analysis. The Lancet. 2008;372(9635):314-20.

12. Baggaley RF, White RG, Boily M-C. HIV transmission risk through anal intercourse: systematic review, meta-analysis and implications for HIV prevention. International journal of epidemiology. 2010;39(4):1048-63.

13. Boily M-C, Baggaley RF, Wang L, Masse B, White RG, Hayes RJ, et al. Heterosexual risk of HIV-1 infection per sexual act: systematic review and meta-analysis of observational studies. The Lancet infectious diseases. 2009;9(2):118-29.

14. Fox J, White PJ, Weber J, Garnett GP, Ward H, Fidler S. Quantifying sexual exposure to HIV within an HIV-serodiscordant relationship: development of an algorithm. AIDS (London, England). 2011;25(8):1065-82.

15. Jin F, Jansson J, Law M, Prestage GP, Zablotska I, Imrie JC, et al. Per-contact probability of HIV transmission in homosexual men in Sydney in the era of HAART. AIDS (London, England). 2010;24(6):907.

16. Vitinghoff E, Douglas J, Judon F, McKiman D, MacQueen K, Buchinder SP. Per-contact risk of human immunodificiency virus tramnsmision between male sexual partners. American journal of epidemiology. 1999;150(3):306-11.

17. Lee R. Occupational transmission of bloodborne diseases to healthcare workers in developing countries: meeting the challenges. Journal of hospital infection. 2009;72(4):285-91.

18. Brogan AJ, Smets E, Mauskopf JA, Manuel SA, Adriaenssen I. Cost effectiveness of darunavir/ritonavir combination antiretroviral therapy for treatment-naive adults with HIV-1 infection in Canada. Pharmacoeconomics. 2014;32(9):903-17.

19. Statistics Canada. Life Tables, Canada, Provinces and Territories 2013 to 201501 June 2018 Available from: <http://www.statcan.gc.ca/pub/84-537-x/84-537-x2018001-eng.htm>.

20. Szende A, Janssen B, Cabases J. Self-reported population health: an international perspective based on EQ-5D: Springer; 2014.
